# The molecular biology of pancreatic adenocarcinoma: translational challenges and clinical perspectives

**DOI:** 10.1038/s41392-021-00659-4

**Published:** 2021-07-05

**Authors:** Shun Wang, Yan Zheng, Feng Yang, Le Zhu, Xiao-Qiang Zhu, Zhe-Fang Wang, Xiao-Lin Wu, Cheng-Hui Zhou, Jia-Yan Yan, Bei-Yuan Hu, Bo Kong, De-Liang Fu, Christiane Bruns, Yue Zhao, Lun-Xiu Qin, Qiong-Zhu Dong

**Affiliations:** 1 Department of General Surgery, Huashan Hospital, Cancer Metastasis Institute, Fudan University, Shanghai, China; 2 Department of Pancreatic Surgery, Pancreatic Disease Institute, Huashan Hospital, Fudan University, Shanghai, China; 3 School of Biomedical Sciences, Li Ka Shing Faculty of Medicine, The University of Hong Kong, Hong Kong, China; 4 General, Visceral and Cancer Surgery, University Hospital of Cologne, Cologne, Germany; 5 Department of Biliary-Pancreatic Surgery, Renji Hospital, School of Medicine, Shanghai Jiao Tong University, Shanghai, China; 6 Department of Surgery, Klinikum rechts der Isar, School of Medicine, Technical University of Munich (TUM), Munich, Germany; 7 Key laboratory of whole-period monitoring and precise intervention of digestive cancer, Shanghai Municipal Health Commission (SMHC), Shanghai, China

**Keywords:** Cancer metabolism, Cancer microenvironment

## Abstract

Pancreatic cancer is an increasingly common cause of cancer mortality with a tight correspondence between disease mortality and incidence. Furthermore, it is usually diagnosed at an advanced stage with a very dismal prognosis. Due to the high heterogeneity, metabolic reprogramming, and dense stromal environment associated with pancreatic cancer, patients benefit little from current conventional therapy. Recent insight into the biology and genetics of pancreatic cancer has supported its molecular classification, thus expanding clinical therapeutic options. In this review, we summarize how the biological features of pancreatic cancer and its metabolic reprogramming as well as the tumor microenvironment regulate its development and progression. We further discuss potential biomarkers for pancreatic cancer diagnosis, prediction, and surveillance based on novel liquid biopsies. We also outline recent advances in defining pancreatic cancer subtypes and subtype-specific therapeutic responses and current preclinical therapeutic models. Finally, we discuss prospects and challenges in the clinical development of pancreatic cancer therapeutics.

## Introduction

Pancreatic cancer, with one of the highest mortality rates of all malignancies, is the seventh leading cause of cancer-related death worldwide.^1^ With almost as many deaths (*n* = 466,000) as new cases (*n* = 496,000) according to GLOBOCAN 2020 estimates,^1^ pancreatic cancer has become the third leading cause of cancer-related death in the United States.^2^ Despite improvements in surgical techniques and therapy regimens, the 5-year survival rate for pancreatic cancer is still the lowest (9%) of any cancer type.^3,4^

Approximately 90% of pancreatic cancers are characterized as pancreatic ductal adenocarcinoma (PDAC). Risk factors associated with pancreatic cancer include familial risk due to susceptibility gene mutations, chronic pancreatitis, pancreatic cysts, and diabetes mellitus.^5,6^ Other risk factors include smoking, alcohol abuse, obesity or metabolic syndrome, aging, and occupational exposure.^4,7^ Approximately 80% of pancreatic cancer patients present with advanced disease or distant metastases and have no effective treatment options.^8^ Even early-stage patients eligible for resection have a 5-year survival rate of <31%.^9^ Advances in next-generation genome sequencing (NGS) have brought new excitement to the field by supporting the identification of molecular alterations that govern pancreatic cancer progression. Sequencing data have revealed that pancreatic cancer comprises highly heterogeneous tumors that develop resistance to traditional chemotherapy and radiation therapy.

Gemcitabine has been a reference first-line therapeutic drug for pancreatic cancer patients since 1997.^10^ However, traditional chemotherapy and radiation therapy have not shown improved 5-year survival rates.^11,12^ Modified FOLFIRINOX (mFOLFIRINOX) (i.e., 5-fluorouracil (5-FU), leucovorin, oxaliplatin, and irinotecan) and nab-paclitaxel plus gemcitabine are generally considered as the best adjuvant chemotherapy regimens, although they have shown only modest survival benefits with considerable toxicity.^13^ Furthermore, multiple novel-targeted therapies (i.e., cetuximab, bevacizumab, axitinib, and aflibercept) have failed to effectively improve overall survival (OS), while erlotinib combined with gemcitabine showed a marginal clinical benefit.^14,15^ Therefore, new therapies, e.g., innovative immunotherapies and combination therapies with increased antitumor potency, are urgently needed.

In this review, we first provide a general discussion of pancreatic cancer. This is followed by a review of novel biomarkers for their diagnosis, treatment monitoring, and prognosis based on liquid biopsy. We conclude with a discussion of the clinical perspectives of current advances. We anticipate that novel therapeutic methods and strategies based on subtype-specific therapy and preclinical therapeutic models might have a great impact on pancreatic cancer treatment and management, thereby improving patient outcomes.

## Characteristics of pancreatic cancer

### Genetics and epigenetics of pancreatic cancer

**Fig. 1:**
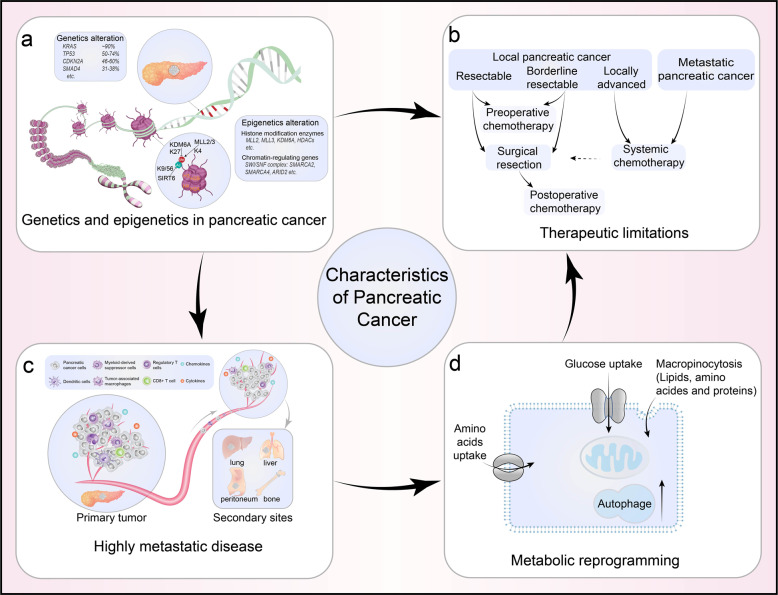
The characteristics of pancreatic adenocarcinoma. Pancreatic cancer is a common cause of cancer mortality characterized by high heterogeneity, a dense stromal tumor microenvironment (TME), highly metastatic propensity, metabolic reprogramming, and limited benefits from current conventional therapies. **a** Genetic and epigenetic changes in pancreatic cancer. *KRAS* (~90%), *TP53* (50–74%), *CDKN2A* (46–60%), and *SMAD4* (31–38%) are the most frequently mutated genes known to modulate the initiation and progression of pancreatic cancer. Epigenetic regulatory genes, including *MLL2/3*, *KDM6A*, and multiple *HDACs* encoding genes, regulate histone modification. *SMARCA2/4* and *ARID2* modulate chromatin remodeling. **b** Therapeutic limitations in pancreatic cancer. Surgical resection is the only potentially curative choice for pancreatic cancer patients. Adjuvant chemotherapy can only partially improve the overall survival of pancreatic cancer patients **c** Pancreatic cancer is an extremely aggressive tumor with high metastatic propensity. The immunosuppressive TME plays an important role in modulating the metastasis of pancreatic cancer cells to the liver, lungs, peritoneum, and bone. **d** Metabolic reprogramming of pancreatic cancer. Pancreatic cancer cells can survive and proliferate in stressful microenvironments by reprogramming energy metabolism to increase glucose and glutamine uptake, macropinocytosis, and autophagy

Via the use of NGS and computational biology, we have gained a deeper understanding of the genetic alterations underlying the genesis and progression of pancreatic cancer, including gene expression changes, copy number aberrations, chromosomal rearrangements, and epigenetic alterations (Fig. 1a). Pancreatic cancer typically starts as a precancerous lesion, i.e., a pancreatic intraepithelial neoplasia (PanIN), which accumulates gene mutations over time, ultimately progressing to a more dysplastic state.^16–18^ Approximately 90% of pancreatic cancers of all grades have activating oncogenic Kirsten rat sarcoma viral oncogene homolog (*KRAS*) mutations. Among the oncogenic *KRAS* mutations associated with pancreatic cancer in humans, the GAT (aspartic acid; G12D), GTT (valine; G12V), and TGT (cysteine; G12C) mutations are the most common, while the CGT (arginine (Arg); G12R) and GCT (alanine; G12A) mutations as well as other point mutations at codons 11, 13, 61, or 146 appear to be less common.^17,18^ As the most common activating mutations in pancreatic cancer, *KRAS* mutations (which have also been investigated in depth in various other cancers, including metastatic colorectal cancer and non-small cell lung cancer) impair the intrinsic GTPase activity of KRAS and prevent GTPase-activating proteins (GAPs) from converting the active GTP-bound form to the GDP-bound inactive form. Clinical studies have also shown that *KRAS* mutations could be considered as a marker for the poor prognosis of pancreatic cancer.^17^ However, it has been reported that various *KRAS* mutations affect diverse signaling pathways, leading to distinct functional consequences. Pancreatic cancer patients with *KRAS* at codon 61 mutations exhibit lower extracellular signal-regulated kinase (ERK) activation compared with patients with other *KRAS* alleles, and the former harbor significantly better prognosis.^19–21^ It is evident that *KRAS* mutation seems to be necessary but not sufficient for pancreatic cancer development. Other genes, including tumor protein p53 (*TP5*3), cyclin-dependent kinase inhibitor 2A (*CDKN2A*), and SMAD family member 4 (*SMAD4*), are also frequently involved in pancreatic cancer tumorigenesis and metastasis.^22^ Approximately 50–74% of pancreatic cancers have inactivating mutations in the tumor suppressor gene *TP53.*^16,23^ *TP53* inactivation impairs DNA damage recognition and blocks cell cycle arrest, allowing cells to bypass cell cycle checkpoints and evade apoptotic signals.^16,23^ Mutations in *CDKN2A*, which can lead to loss of regulation of the cyclin-dependent kinase (CDK) 4 and CDK6 cell cycle checkpoints and, thus, dysregulation of the cell cycle and subsequent carcinogenesis, are detected in ~46–60% of pancreatic cancers.^16,23,24^ In addition, ~31–38% of pancreatic cancers have mutations in *SMAD4*, which can occur in the late stages of pancreatic carcinogenesis.^23,25^ In pancreatic cancer, the frequent loss of *SMAD4* via homozygous deletion or mutation leads to decreasing SMAD4-dependent inhibition of transforming growth factor-β (TGF-β) and the promotion of noncanonical TGF-β signaling, thereby facilitating pro-tumorigenic responses.^23,26,27^ Recently, high-throughput sequencing studies have revealed genes with novel mutations/alterations with individual frequencies <20%, including lysine demethylase 6A (*KDM6A*) (18%), rac family small GTPase 1 (*RAC1*) (10%), ring-finger protein 43 (*RNF43*) (10%), AT-rich interaction domain 1A (*ARID1A*) (9%), B-Raf proto-oncogene, serine/threonine kinase (*BRAF*) (3%), TGF-β receptor 2 (*TGFBR2*) (3%), mitogen-activated protein kinase kinase kinase 21 (*MAP3K21*) (3%), switch/sucrose nonfermentable (SWI/SNF)-related, matrix-associated, actin-dependent regulator of chromatin, subfamily a, member 4 (*SMARCA4*) (3%), activin A receptor type 2A (*ACVR2A*) (2%), activin A receptor type 1B (*ACVR1B*) (2%), N-ras proto-oncogene, GTPase (*NRAS*) (1%), family with sequence similarity 133 member A (*FAM133A*) (<1%), and zinc-finger matrin-type 2 (*ZMAT2*) (<1%).^24,28,29^ It has been reported that 4–7% of pancreatic cancer patients have a germline DNA repair associated (*BRCA*) mutation.^30^ Furthermore, germline BRCA2 mutations have been observed in 5–17% of familial pancreatic cancer patients.^31^ Mutation of the GNAS complex locus (*GNAS*) at codon 201 has been observed exclusively in intraductal papillary mucinous neoplasms (IPMNs), a precursor to pancreatic cancer, at a rate of 41–75%.^32^ However, several studies have reported that ~4% of pancreatic cancer patients have *GNAS* mutations.^24^ Most of these low-frequency mutations are in genes related to cellular processes such as cell survival, cell fate determination, and genome maintenance, involving various signaling pathways.^28,33^ In addition, recurrent noncoding mutations, which are enriched in transcriptionally active regions of the genome, also play an essential role in pancreatic cancer.^34^

Epigenetic alterations, which can regulate histone or DNA modification, play significant roles in molecular aspects of pancreatic tumorigenesis.^35^ In pancreatic cancer, epigenetic regulatory genes are frequently mutated, including those encoding SWI/SNF-mediated chromatin remodeling complexes and histone modification enzymes.^36^ Commonly mutated histone modification enzymes include the mixed-lineage leukemia (MLL) histone methylases (MLL2 and MLL3), histone methyltransferases, and the KDM6A histone demethylase.^25,37^ Furthermore, MLL2/3 and KDM6A exist in the same complex, which drives transcriptional activation through coordinated regulation of histone 3 lysine 4 (H3K4) methylation and histone 3 lysine 27 (H3K27) demethylation.^38^ During pancreatic cancer development, tumors with genetic defects in MLLs are more likely to induce expression of chromatin-regulating genes and cell proliferation-associated genes (including members of the SWI/SNF chromatin remodeling complex) as well as genes involved in cell cycle progression and cellular proliferation.^37^ The SWI/SNF complexes comprise multiple components encoded by different genes, including SWI/SNF-related, matrix-associated, actin-dependent regulator of chromatin, subfamily a, member 2 (*SMARCA2*), *SMARCA4*, *SMARCE1*, *SMARCB1*, AT-rich interaction domain 2 (*ARID2*), *ARID1A*, AT-rich interaction domain 1B (*ARID1B*), and double PHD fingers 2 (*DPF2*). The overall prevalence of gene mutations or copy number variations in SWI/SNF complex components is >10%; thus, such alterations have important effects on chromatin remolding in pancreatic cancer pathogenesis.^20,28,36^ Histone deacetylases (HDACs), which catalyze histone deacetylation, are classified into two enzyme subtypes: the NAD^+^-dependent sirtuins (SIRT1–7) and the zinc-dependent HDAC1–11 proteins.^39^ Interestingly, HDAC1 and HDAC2 are highly expressed in pancreatic cancer, and they can be recruited to the epithelial-cadherin (CDH1) promoter (which is involved in histone deacetylation) by zinc-finger E-box binding homeobox 1 (*ZEB1*) or Snail, thereby promoting epithelial-to-mesenchymal transition (EMT) and tumor metastasis.^40,41^ 4SC-202, a specific inhibitor of class I-HDACs, can inhibit TGF-β-induced EMT and p21 cyclin-dependent kinase inhibitor 1A (*CDKN1A*)-mediated cell proliferation.^42^ SIRT6 ablation can potently cooperate with activated KRAS to promote pancreatic cancer metastasis via the hyperacetylation of histone 3 lysine 9 (H3K9) and histone 3 lysine 56 (H3K56) at the promoter of the lin-28 homolog B (*LIN28b*) gene, resulting in Myc recruitment and Lin28b upregulation. As a result, the expression levels of high-mobility group AT-hook 2 (*HMGA2*), insulin-like growth factor 2 mRNA-binding protein (*IGF2BP*) 1, and *IGF2BP3*, which are downstream of target genes let-7, are increased.^43^

### Subtyping of pancreatic cancer

Pancreatic cancer is a deadly malignancy that lacks effective therapeutics (Fig. 1b). This is in part due to the inter- and intratumor heterogeneity.^44–46^ Pancreatic cancer molecular subtype identification has the potential to improve clinical outcomes by allowing the development of individualized treatments. Recently, various molecular subtypes and subtype-specific treatment responses in pancreatic cancer have been recognized.^36,44,47^ The two predominant transcriptomic-based subtypes, which have been validated across multiple studies, are the classical/pancreatic progenitor subtype and the basal-like/squamous/quasi-mesenchymal (QM-PDA) subtype.^44,46,47^ On the other hand, the existence of the exocrine-like/aberrantly differentiated endocrine exocrine (ADEX) subtype and the immunogenic subtype, which were proposed by Collisson et al. and Bailey and co-workers, have been controversial.^44,47,48^ Due to the desmoplastic nature of pancreatic cancer, low tumor cellularity remains a major concern in sampling and defining the molecular subtypes of this disease. Collisson et al. identified classical and QM-PDA subtypes within a large panel of pancreatic cancer cell lines, but did not detect an exocrine-like subtype. The Cancer Genome Atlas (TCGA) project found that the ADEX subtype and immunogenic subtype were associated with low cellularity, likely representing non-transformed cells.^49^ Later, Puleo et al. confirmed that the ADEX tumor subtype resulted from contamination of their pancreatic exocrine and endocrine components with adjacent normal pancreatic acinar cells.^48^ However, Noll et al. and Knudsen et al. identified the exocrine subtype in their patient-derived pancreatic cancer models.^50,51^ In addition, the exocrine subtype showed resistance to tyrosine kinase inhibitors and paclitaxel treatment via the expression of cytochrome P450 family 3 subfamily A member 5 (*CYP3A5*).^50^ A recent study reported significant variation in the tissue composition of pancreatic tumors from the Moffitt, Bailey, and TCGA cohorts. Up to 45% of the tumor samples from various public cohorts were reclassified after removal of the non-epithelial signal from the tumor bulk expression profiles.^52^

### Signaling pathways regulating pancreatic cancer tumorigenesis and metastasis

Signaling pathways (e.g., RAS, phosphoinositide 3-kinase (PI3K)/protein kinase B (AKT), nuclear factor kappa-light-chain-enhancer of activated B cells (NF-κB), janus kinase (JAK)/signal transducer and activator of transcription (STAT), Hippo/yes-kinase-associated-protein (YAP), Wingless/int1 (WNT), etc.) have been linked to a variety of cancer-related cellular processes, including cell proliferation, differentiation, apoptosis, migration, angiogenesis, metabolism, and immune regulation. They have been implicated in pancreatic cancer tumorigenesis, prognosis, and resistance to therapy, it is likely that a deep understanding of these pathways will support the development of molecularly targeted pancreatic cancer therapies.

#### RAS pathway

RAS signaling is centered on the activated small GTPase RAS, which drives the activation of three major effector pathways, RAS-like proto-oncogene A/B, RAF/MAP kinase (MEK)/ERK, and PI3K. Oncogenic activation of RAS due to missense mutations is frequently detected in several types of cancer, including pancreatic cancer.^53^ Given that mutations in one RAS protein isoform, KRAS, are found in nearly 90% of pancreatic cancers, RAS signaling appears to play a critical role in both pancreatic cancer initiation and maintenance. Activated RAS can activate effector signaling pathways and transcription factors involved in cell transformation, proliferation, and metastasis. Activated RAS also promotes pro-inflammatory signaling via activation of NF-κB, signal transducer and STAT3, and glycogen synthase kinase-3/nuclear factor of activated T cell signaling.^54–56^ Pancreatic cancers without *KRAS* mutations show RAS activation via upstream signaling through receptor tyrosine kinases (RTKs), e.g., epidermal growth factor receptor (EGFR), and oncogenic activation of the downstream B-Raf proto-oncogene (B-RAF) molecule is detected in a small number of patients.^57^ Despite extensive insight into the molecular mechanisms of KRAS in multiple cancer-promoting effects, it has been challenging to obtain clinically effective KRAS inhibitors, with the exception of a KRAS G12C (carried by ~1.5% of pancreatic cancer patients)-selective inhibitor AMG 510.^58^ In mouse models, KRAS inhibition can cause the activation of AKT, erb-b2 RTK2 (HER2), platelet-derived growth factor receptor alpha, and EGFR, which might explain the inefficacy of these inhibitors.^28^

#### PI3K/AKT pathway

Since PI3K can be activated through an initial phosphorylation in response to oncogenic RAS, the PI3K/AKT pathway is frequently activated in human pancreatic cancer and mouse models of KRAS-driven pancreatic cancer.^28,59^ Abnormal overexpression or activation of AKT is associated with >40% of pancreatic cancer patients. Abnormal AKT overexpression or activation was also reported to be associated with short survival and the pathologic grade of pancreatic cancer.^60,61^ It was demonstrated that PI3K/AKT activation appears to occur at the earliest stages of tumor evolution and that it controls pancreatic cell plasticity and carcinogenesis, as strong activation of PI3K signaling was observed in human acinar–ductal metaplasia (ADM) and in both low- and high-grade PanIN.^62^ Interestingly, PI3K p110α is required for pancreatic cell plasticity and cancer initiation induced by oncogenic *KRAS*,^63^ and constitutively active AKT1 cooperates with active KRAS (G12D) in the AKT pathway to accelerate pancreatic tumor onset and progression.^64^ Insulin-like growth factor (IGF) has been demonstrated to enhance the invasion and proliferation of pancreatic cancer cells via activation of the PI3K/AKT signaling pathway.^65^ Our recent study demonstrated that the PI3K/AKT/mTOR pathway regulates enolase 2 (ENO2) K394 deacetylation upon IGF-1 stimulation, thereby promoting liver metastasis in pancreatic cancer.^66^ Abnormal overexpression of *IGF2BP2* caused by genomic amplification or posttranscriptional regulation promotes pancreatic cancer proliferation by activating the PI3K/AKT signaling pathway.^67^ In addition, multiple noncoding RNAs (ncRNAs), including miR-107, lncRNA ABHD11-AS1, lncRNA SNHG1, lncRNA AB209630, and transmembrane protein 158 have been reported to govern tumorigenesis, progression, metastasis, apoptosis, or drug resistance in pancreatic cancer cells via the PI3K/AKT pathway.^68,69^ Currently, several PI3K/AKT inhibitors are under investigation, but none are in routine clinical use. Some clinical trials of PI3K/AKT inhibitors (alone or in combination with chemotherapy drugs) in pancreatic cancer patients might reveal promising therapeutic effects.

#### NF-κB pathway

Constitutive activation of NF-κB, a transcription factor that is essential for inflammatory responses, has been frequently observed in pancreatic cancer. Increasing evidence suggests that both canonical and noncanonical NF-κB pathways can affect pancreatic cancer progression, metastasis, and drug resistance.^70,71^ *KRAS* mutation as well as oncogenic mutations in other genes, e.g., *EGFR*, *PI3K*, and *TP53*, also contribute to NF-κB activation in pancreatic cancer.^72^ Elevated levels of inflammatory cytokines and chemokines are observed in pancreatic cancer cells, and these increased levels are generally associated with the enhanced NF-κB signaling.^73^ For example, activated NF-κB translocates into the nucleus to enhance transcription of downstream inflammatory target genes, including interleukin-6 (IL-6), IL-8, and IL-18, and increased levels of these cytokines lead to the activation of NF-κB signaling, thereby forming a positive feedback loop.^74,75^ Several studies have revealed that ncRNAs regulate the NF-κB signaling pathway by directly interacting with the functional domain or its transcripts. Overexpression of lncRNA-PLACT1 promotes pancreatic cancer progression via constitutive activation of NF-κB signaling. MiR-628-5p functions as a tumor-suppressive microRNA (miRNA) in pancreatic cancer by negatively regulating phospholipid scramblase 1 and insulin receptor substrate 1 expression, which inhibits NF-κB signaling.^76^ Furthermore, NF-κB is reported to be involved in antitumor immunity. NF-κB in pancreatic stromal cells (PSCs) contributes to tumor growth by increasing the expression of C–X–C motif chemokine ligand 12 (CXCL12), which prevents cytotoxic T cells from infiltrating the tumor and killing cancer cells.^77^ NF-κB is required for pancreatic cancer cells to evade macrophage surveillance, and growth differentiation factor 15-mediated inhibition of NF-κB signaling in infiltrating macrophages blocks the antitumor immune response during the early stages of tumorigenesis.^78^ A variety of NF-κB pathway inhibitors, including small molecules, peptides, small DNA/RNAs, viral proteins, and natural compounds, despite not being tested specifically in a pancreatic cancer model, have shown great promise.^79^

#### JAK/STAT pathway

The JAK/STAT pathway is clearly involved in many types of human cancer, including pancreatic cancer. High JAK2 expression predicts a poor prognosis in patients with PDAC.^80^ Several studies have shown that the JAK/STAT pathway is involved in inflammatory processes in pancreatic cancer. Both type 1 (i.e., IFNα and IFNβ) and type 2 (i.e., IFNγ) interferons can upregulate programmed cell death 1-ligand 1 (PD-L1) expression via the JAK-STAT signaling pathway in pancreatic cancer.^81,82^ Sustained JAK/STAT pathway activation mediates chronic inflammation and impairs cytotoxic T lymphocyte (CTL) activation in pancreatic cancer, and the JAK-STAT inhibitor ruxolitinib can increase CTL infiltration to induce a Tc1/Th1 immune response in the tumor microenvironment of pancreatic cancer.^83^ In the PDAC microenvironment, it was demonstrated that tumor-derived IL-1 induces LIF expression and downstream JAK/STAT activation to generate inflammatory cancer-associated fibroblasts (iCAFs), whereas tumor-derived TGF-β antagonizes this process by downregulating IL-1 receptor type 1 expression and promoting differentiation into myofibroblasts (myCAFs).^84^

#### Hippo/YAP pathway

YAP and its transcriptional coactivator with PDZ-binding motif (TAZ) are the two major downstream effectors of the Hippo pathway.^85^ Several studies have revealed that YAP is upregulated in pancreatic cancer patients, and YAP overexpression is correlated with liver metastasis and poor prognosis of pancreatic cancer.^86^ YAP has been reported to be insufficient to drive an initial step in the progression to pancreatic cancer with ADM. However, YAP is required for the induction of ADM progression to PanIN, and PanIN progression to pancreatic cancer.^87,88^ Accumulating studies have demonstrated that YAP is a critical player in pancreatic cancer progression in KRAS mutant mice.^88^ Active YAP promotes pancreatic tumor tumorigenesis, development, metastasis, stromal response, drug resistance, and metabolic homeostasis in KRAS-driven pancreatic cancer.^89,90^ However, it has been demonstrated that YAP is sufficient to drive PDAC recurrence in the absence of KRAS via bypass mechanisms involving YAP.^91^ Recent studies have also demonstrated that YAP is a major driver of the squamous subtype of pancreatic cancer, which is notably less dependent on oncogenic KRAS.^92^ These findings indicate that YAP not only acts as a pancreatic cancer driver downstream of KRAS but that it also substitutes for loss of oncogenic *KRAS.*^93^ Furthermore, YAP was identified as a critical regulator of the immunosuppressive microenvironment. YAP and TAZ can modulate the behavior of pancreatic stellate cells (PSCs) and influence the recruitment of tumor-associated macrophages (TAMs) and myeloid-derived suppressor cells. For example, YAP has been shown to promote the expression and secretion of multiple cytokines and chemokines, which in turn promote the differentiation and accumulation of myeloid-derived suppressor cells (MDSCs) in pancreatic cancer.^94–96^

#### WNT pathway

The WNT signaling pathway includes the canonical and noncanonical pathways and controls the maintenance of somatic stem cells in many tissues. Both the canonical (β-catenin-dependent) and noncanonical (β-catenin-independent) pathways have been implicated in pancreatic carcinogenesis, tumor progression, and therapeutic resistance.^97,98^ WNT signaling is required for KRAS-induced PanIN lesions and pancreatic cancer formation.^99^ KRAS activation can promote the invasion and migration of pancreatic cancer cells by regulating the WNT/β-catenin signaling pathway or by increasing the interaction between β-catenin and cyclic AMP-response element-binding protein-binding protein.^100^ Increased WNT/β-catenin signaling activation results in an enhanced stem cell-like phenotype of pancreatic cancer.^101^ Activation of the canonical pathway can prevent β-catenin degradation and promote its nuclear translocation, which enhances the transcription of targeted genes such as *cyclin D1* and *c-Myc*.^102,103^ Aberrant nuclear accumulation of β-catenin is frequently found in PanIN and pancreatic cancer and is associated with their development. Canonical WNT ligands, such as Wnt family member 2, Wnt family member 5A, and Wnt family member 7A, have been observed to be increased in pancreatic cancer tissues, and activated the WNT pathway, leading to the progression of pancreatic cancer.^104,105^ In addition, noncanonical ligands, such as mucin (MUC) family members (MUC1 and MCU4) and R-spondin, activate the WNT pathway, leading to pancreatic cancer progression.^106–108^ Pancreatic cancer is characterized by hypoxic conditions. Hypoxia-inducible factor-2α (HIF-2α) modulates WNT signaling by maintaining the levels of both SMAD4 and β-catenin during PanIN progression.^109^ Moreover, hypoxic conditions in pancreatic tumors stabilize HIF-2α, which interacts with β-catenin, leading to elevated canonical WNT/β-catenin activity while favoring tumor progression.^110^

### High invasion and metastasis of pancreatic cancer

Pancreatic cancer is generally considered an extremely aggressive tumor with high metastatic propensity. Most patients are diagnosed with advanced metastatic disease with a dismal prognosis. A better understanding of the mechanisms underlying metastatic progression is required to develop improved therapeutic interventions.^111^

It has been demonstrated that metastasis can occur during the early stages of pancreatic cancer, even before large mass formation by the primary tumor^112,113^ (Fig. 1c). It has been conceptualized that a stepwise accumulation of genetic and epigenetic alterations is the driving force for this process.^114^ Previous efforts to identify a signature of prometastatic genes or a consistent pattern of metastasis-driving mutations via genomic sequencing of human primary pancreatic tumors and their metastatic sites have failed.^115^ Nevertheless, distant metastatic lesions are hypothesized to evolve from the original tumor cells by acquiring distinct gene mutations.^116^

To metastasize, neoplastic cells must detach from the primary tumor and travel through the blood vessels or lymphatic system. A number of ncRNAs,^117,118^ transcription factors (e.g., Kruppel-like factor 4 (KLF4),^119^ KLF5,^120^ KLF7^121^), growth factors (e.g., vascular endothelial growth factor (VEGF)^122^), and oxygen conditions^123^ have been reported to drive pancreatic cancer metastasis. Using an organoid culture system, Roe et al. studied how transcription and the enhancer landscape evolves during the metastatic transition in a pancreatic cancer mouse model. They found that forkhead box A1-dependent enhancer reprogramming can promote the acquisition of metastatic traits.^124^ In pancreatic cancer, the most common metastatic site is the liver, followed by the lungs, peritoneum, and bones.^125–127^ Recently, Lee et al. reported that activation of STAT3 in hepatocytes and the subsequent production of serum amyloid A1 and A2 in the bloodstream direct pancreatic cancer liver metastasis.^128^ Interestingly, lineage-tracing analysis in a mouse model of pancreatic cancer revealed that metastases in the lung and liver are monoclonal, whereas those in the peritoneum and diaphragm tend to be polyclonal. These findings indicate that clonal diversity depends on the metastatic site.^129^ Global gene expression and molecular profiling studies showed that cytokines (e.g., IL-8), growth factors (e.g., hepatocyte growth factor and VEGF), and matrix metalloproteinase (e.g., extracellular matrix (ECM) protease)^122,130,131^ as well as membrane receptors were key differentiating factors underlying pancreatic cancer-derived hematogenous metastasis vs. peritoneal dissemination.^127^ In pancreatic cancer, lymph node metastasis is considered a critical risk factor in patients with high-risk features.^132^ By using data and tumor samples from three independent cohorts, a recent study identified a miRNA signature associated with pancreatic cancer patients at risk for lymph node metastasis.^133^ Additional evidence has demonstrated that extracellular vesicles (EVs), such as exosomes, are essential for pancreatic cancer initiation and metastasis.^134^ Pancreatic cancer cell-derived exosomes can be taken up by Kupffer cells, resulting in TGF-β secretion and upregulation of fibronectin production by hematopoietic stem progenitor cells, which then induce liver pre-metastatic niche formation.^135^ Moreover, exosome-derived integrin (α6β4, α6β1, and αvβ5) uptake by resident cells (liver Kupffer cells, lung fibroblasts, or epithelial cells) contributes to organ-specific pre-metastatic niche formation via activation of SRC proto-oncogene (Src) phosphorylation and pro-inflammatory *S100* gene expression.^136^ The extensively studied metastatic process of EMT, which represents the transition of epithelial cells into mesenchymal cells, has been shown to be pivotal during pancreatic cancer cell invasion and metastasis.^137–139^ TGF-β was the first identified cytokine that induced EMT in pancreatic cancer cells via the RAS-MEK-ERK signaling pathway.^140^ Subsequent studies have demonstrated that EMT in pancreatic cancer cells can be triggered by various factors, including growth factors, cytokines, transcription factors, and miRNAs.^141,142^ Our group found that IGF-1 induced ENO2 deacetylation by HDAC3-enhanced EMT, thus promoting liver metastasis of pancreatic cancer.^66^ In addition, pancreatic cancer cells undergoing EMT can induce a cancer stem cell (CSC) phenotype as well as drug resistance.^143,144^

The pancreatic tumor microenvironment is indispensable for pancreatic cancer progression. Reciprocal communication between cancer cells and stromal cells induces changes in cellular components of the pancreatic tumor microenvironment, which can prime the primary tumor for metastasis and cell migration.^145^ Primary pancreatic tumors can modulate the local microenvironment in metastatic sites by secreting exosomes and soluble factors to promote colonization.^146,147^ Various stromal cells, e.g., TAMs, also participate in angiogenesis and metastasis of pancreatic cancer via the secretion of VEGF, CXCL1, and CXCL8.^148,149^

## Metabolism dysregulation in pancreatic cancer

**Fig. 2:**
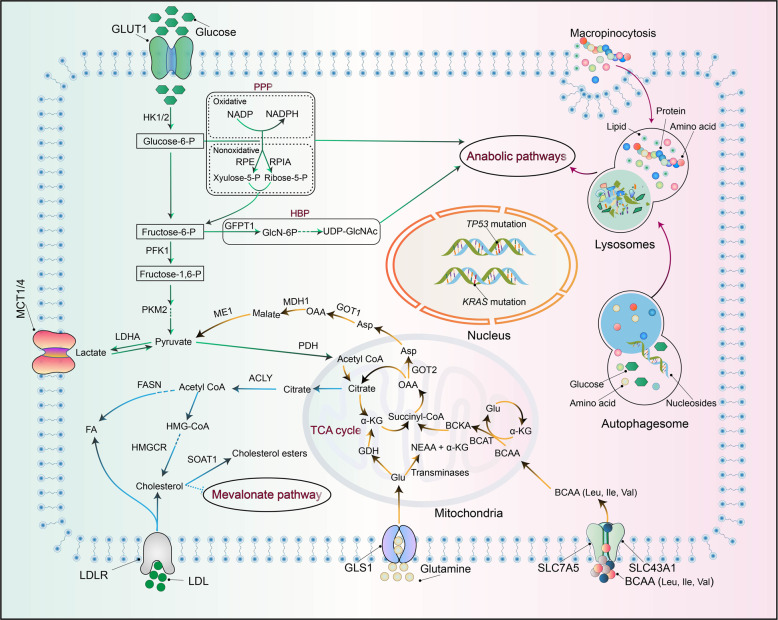
Metabolic reprogramming in pancreatic cancer cells. *KRAS* activation and mutant *TP53* enhance glucose metabolism to provide biosynthetic precursors for anabolic pathways, including the non-oxidative arm of the pentose phosphate pathway (PPP) and the hexosamine biosynthesis pathway (HBP). KRAS activation reprograms glutamine metabolism to sustain cellular redox homeostasis by increasing the NADPH/NADP^+^ ratio and recycling glutathione (GSH) via reduction of oxidized GSH. The BCAT2-mediated BCAA catabolism driven by KRAS plays a critical role during pancreatic cancer development. Enhanced nutrient salvaging, via the induction of macropinocytosis and autophagy, provides energy and regenerative nutrients, including glucose, amino acids, lipids, and nucleosides

Metabolic reprogramming is a hallmark of cancer, and it influences the survival and growth of cancer cells by providing energy and macromolecular precursors.^150^ Pancreatic cancer is characterized by hypovascularization, and pancreatic cancer cells are surrounded by a tight desmoplasia, thus creating a highly hypoxic and nutrient-limited microenvironment.^151,152^ Malignant pancreatic cancer cells proliferate uncontrollably and have a proclivity for distant metastasis, which results in increased demand for both energy and biosynthetic precursors (Fig. 1d).^153^ Metabolic reprogramming is central to the pathogenesis of pancreatic cancer driven by KRAS (Fig. 2). A recent study reported that pancreatic cancer tumors can be classified into four metabolic subgroups, i.e., quiescent, glycolytic, cholesterogenic, and mixed metabolic profiles, based on the expression levels of glycolytic and cholesterogenic genes. Glycolytic tumors correlate with poor outcomes in pancreatic cancer patients, whereas patients with cholesterogenic tumors tend to have better survival, possibly due to their higher energy expenditure.^154^

### Reprogrammed glucose metabolism in pancreatic cancer

Cancer cells can survive and proliferate in stressful microenvironments by reprogramming their energy metabolism to increase glucose uptake and enhance glycolysis and lactate production despite the presence of oxygen (known as the Warburg effect).^155^ Notably, pancreatic cancer cells exhibit extensive glucose metabolic reprogramming, including glycolytic enzyme overexpression and increased lactate production.^152,156^ It has been demonstrated that oncogenic *KRAS* promotes glycolysis by enhancing glucose uptake via upregulation of the glucose transporter (GLUT1) and several rate-limiting glycolytic enzymes, including hexokinase 1 and 2, phosphofructokinase-1, and lactate dehydrogenase A.^156,157^ Furthermore, in pancreatic cancer cells, mutant *KRAS* signaling promotes mitochondrial translocation of phosphoglycerate kinase-1, resulting in the production of phosphorylated pyruvate dehydrogenase kinase-1 and restricted oxidative phosphorylation (OXPHOS).^158^ Enhanced glycolysis induced by oncogenic *KRAS* provides biosynthetic precursors for anabolic pathways, including the hexosamine biosynthesis pathway (HBP) and the non-oxidative arm of the pentose phosphate pathway (PPP).^156^ *KRAS* mutations drive the HBP, a side path of glycolysis, to increase the generation of biosynthetic precursors required for protein glycosylation and the synthesis of proteoglycans, glycolipids, and glycosyl phosphatidylinositol anchors.^156,159^ This process depends on both increased glycolysis and transcriptional upregulation of glutamine (Gln) fructose 6-phosphate transamidase 1 in the HBP.^156^ Similarly, oncogenic *KRAS* enhances the flux of glucose-derived carbon into the non-oxidative arm of the PPP to generate ribose-5-phosphate for de novo nucleotide biosynthesis, thus fueling proliferation.^160^ In addition, p53 also plays a vital role in metabolic reprogramming in pancreatic cancer cells. It has been demonstrated that KRAS and p53 can promote metabolic changes even before the occurrence of malignant transformation.^161^ In pancreatic cancer cells, mutant *TP53* can activate GLUT1-mediated glucose transport by upregulating paraoxonase 2 expression and impairing the expression of *TP53*-induced glycolysis regulatory phosphatase to promote glycolysis.^162^ A gain-of-function *TP53* allele was demonstrated to enhance the Warburg effect by inducing GLUT1 translocation to the plasma membrane, which is regulated by the RAS homolog family member A/rho-associated coiled-coil containing protein kinase/GLUT1 signaling pathway.^163^ In addition, *TP53* mutations can decrease mitochondrial activity to suppress pancreatic cancer progression.^164^ Highly hypoxic microenvironments commonly form during pancreatic cancer progression, thus increasing the expression and stability of HIF-1. The hypoxic condition in the tumor microenvironment and HIF-1α upregulation contributes to elevated glycolytic activity and these factors cooperate with mutant *KRAS* to sustain cytosolic ATP generation even though knockdown of its expression has limited influence on metabolism-related enzyme expression.^156,165^ HIF-1α upregulation has been shown to upregulate GLUT1 as well as the expression levels of glycolysis-related genes to sustain cytosolic ATP levels in pancreatic cancer cells.^166,167^ HIF-1α also upregulates the expression of GFPT2 and inhibits the expression of pyruvate dehydrogenase to restrict mitochondrial oxidation.^168^

### Reprogrammed amino acid metabolism in pancreatic cancer

It has been demonstrated that amino acid metabolism plays a crucial role in pancreatic cancer progression.^169,170^ Among the amino acids, Gln is the most abundant in circulation and is a major source of carbon and nitrogen for cancer cells.^171^ Notably, Gln is essential to support pancreatic cancer growth via redox homeostasis in a KRAS-driven metabolic pathway.^156,172,173^ Inhibition of downstream components of Gln metabolism leads to decreased tumor growth.^151,172^ Modification of the activity of glutaminase (GLS), which catalyzes the first step in glutaminolysis, i.e., the conversion of Gln to glutamate (Glu) in mitochondria, represents a viable therapeutic strategy. Interestingly, pancreatic cancer cells exhibit compensatory metabolic networks that sustain progression after GLS inhibition.^174^ When GLS1 was inhibited in pancreatic tumors, the GLS2 pathway was upregulated for Glu production.^175^

Arg can be directly catalyzed by oxide synthases (NOS1–3) into citrulline and nitric oxide (NO). NO promotes pancreatic cancer cell proliferation, and this effect can be dampened by knocking down NOS3 activity in *KRAS* mutant tumors.^176^ Arg deprivation is currently being evaluated for safety and efficacy in clinical trials. A recent study reported that Arg deprivation inhibits pancreatic cancer cell migration, invasion, and EMT.^177^ The mammalian target of rapamycin complex 1 (mTORC1) kinase is a master growth regulator that senses many environmental cues, including amino acid levels.^178,179^ Via solute carrier family 38 member 9, Arg serves as a lysosomal messenger that couples mTORC1 activation to the lysosomal release of essential amino acids needed to drive pancreatic cancer cell growth.^179,180^

In addition, proline dehydrogenase (PRODH)/proline oxidase, which catalyzes the first step of proline degradation, has been extensively linked to the progression of cancer, including pancreatic cancer.^181^ PRODH1 expression is elevated in pancreatic cancer cells to sustain cellular survival and proliferation under low-glucose or Gln-limited conditions.^182^ Via experiments with genetically engineered mouse models (GEMMs) and primary pancreatic epithelial cells, a recent study demonstrated that induction of the serine–glycine one-carbon pathway plays a vital role in tumorigenesis.^183^ In recent years, branched-chain amino acid (BCAA) metabolism has been tentatively linked to pancreatic cancer development.^184^ As essential amino acids, BCAA must be derived from the extracellular milieu, and specific transporters responsible for BCAA (isoleucine, leucine, and valine) absorbance include members of the solute carrier family 7 (SLC7) family (SLC7A5A and SLC7A8) and the solute carrier family 43 (SLC43) family (SLC43A1 and SLC43A2).^185,186^ It has been reported that plasma BCAAs are elevated in patients with increased pancreatic cancer risk and in mice with early-stage pancreatic cancer driven by mutant *KRAS.*^187^ BCAA transaminase 2 (BCAT2), a BCAA transaminase, is upregulated in advanced pancreatic cancer.^188^ A recent study demonstrated that BCAT2 plays a central role in the development of ductal-derived PanIN lesions by sustaining BCAA catabolism, and BCAT2-mediated BCAA catabolism driven by KRAS is critical for pancreatic cancer development

### Reprogrammed lipid metabolism in pancreatic cancer

Lipid metabolism is an essential cellular process that converts nutrients into metabolic intermediates for the maintenance of cellular structures, energy storage, and the generation of signaling molecules production. It is increasingly reported that dysregulation of lipid metabolism is associated with the progression of various cancers, including pancreatic cancer.^189,190^

In mice, a high-fat diet can increase oncogenic *KRAS* activity, leading to fibrosis, inflammation, and enhanced PDAC development.^191^ It has been demonstrated that multiple enzymes that catalyze de novo fatty acid (FAs) synthesis, including ATP citrate lyase, FA synthase (FASN), and stearoyl-CoA desaturase, are obviously upregulated in pancreatic cancer.^192,193^ In addition, increased FASN expression was correlated with poor survival and poor gemcitabine responsiveness through enhanced estrogen receptor stress and cancer stemness. Inhibition of FA biosynthesis could be a promising strategy to overcome gemcitabine resistance in pancreatic cancer.^194^ Interestingly, different FAs might play different roles in pancreatic cancer. Saturated FAs promote tumor progression by increasing cyclooxygenase-2, VEGF, and caveolin 1 expression and the production of lipid droplets (LDs). High-fat diets enriched in monounsaturated FAs and ω6 polyunsaturated FAs (PUFAs) can increase tumor size in pancreatic cancer mouse models, and ω6 PUFA-rich diets frequently lead to liver metastasis by increasing hepatic ω6 PUFAs levels.^195^ ω3 PUFAs decrease pancreatic cancer cell proliferation by reducing AKT phosphorylation, while ω6 PUFAs can promote tumor growth by increasing AKT phosphorylation.^196^ FA β-oxidation (FAO) is essential for energy maintenance to support the proliferation and survival of cancer cells.^197,198^ A recent study demonstrated that the main source of ATP production in pancreatic cancer depends on FAO rather than glycolysis.^199^ Excess lipids stored as LDs can be converted into FAs by adipose triglyceride lipase and hormone-sensitive lipase (HSL) to provide ATP for pancreatic cancer metastasis. A recent study demonstrated that oncogenic mutant *KRAS* in pancreatic cancer facilitated LD utilization via suppressing the expression and phosphorylation of HSL, thereby fueling cancer cell invasion.^200,201^

In an oncogenic KRAS mouse model, the comparative transcriptomic analysis identified lipid-related metabolic pathways, in particular cholesterol uptake, as being the most highly enriched in pancreatic cancer (compared with a normal pancreas).^192^ Pancreatic cancer patients with increased expression of cholesterol synthesis genes showed a survival benefit in resectable and metastatic cases.^154^ Furthermore, cholesterol metabolism plays an important role in controlling pancreatic cancer development and differentiation. By using a GEMM driven by *KRAS*^G12D^ mutation and homozygous *TP53* loss, a recent study demonstrated that inhibition of cholesterol biosynthesis with statins, a widely used inhibitor of the mevalonate pathway, or NAD(P)-dependent steroid dehydrogenase-like enzyme inhibitor can switch glandular pancreatic carcinomas to a basal phenotype.^202^ Loss of function of *TP53* can drive tumorigenesis via downregulation of cholesterol transporter gene ATP-binding cassette subfamily A member 1 and activation of PI3K/sterol regulatory element-binding protein 2 (SREBP2) maturation. Consequently, the mevalonate pathway, which is responsible for cholesterol and sterol biosynthesis, is upregulated.^203,204^ Statins can modestly affect cholesterol homeostasis and significantly reduce the synthesis of electron carrier coenzyme Q, leading to severe oxidative stress and apoptosis in pancreatic cancer.^205^ By using organoid and mouse models, a recent study demonstrated that the loss of sterol O-acyltransferase 1 (SOAT1), a key enzyme catalyzing cholesterol to inert cholesterol esters to sustain mevalonate pathway flux, significantly impairs pancreatic cancer progression. Mechanistically, mutant *TP53* and *TP53* LOH can promote the expression of SOAT1, which upholds SREBP2-driven cholesterol biosynthesis to sustain the levels of multiple nonsterol isoprenoids, e.g., farnesyl pyrophosphate and geranylgeranyl pyrophosphate, ultimately resulting in RAS and Rho activation. These findings indicate that SOAT1 inhibition might be a potential therapeutic modality for pancreatic cancer patients with mutant *TP53* and *TP53* LOH.^206^

### Autophagy and nutrient salvage regulation in pancreatic cancer

Autophagy, a cellular “self-eating” process, allows cells to degrade and recycle their own cellular components, thereby enhancing their survival under stress conditions. Notably, it seems that autophagy has conflicting functions during cancer progression, including both tumor-suppressive and tumor-promoting effects, which are likely context-dependent. The role of autophagy in pancreatic cancer is also complex. During early stages, autophagy is antitumorigenic because of its function in cellular quality control, while in established cancer, autophagy can support cancer cell survival and progression by providing resources for macromolecule biosynthesis and bioenergetics.^207^ A growing body of evidence suggests that autophagy is required for pancreatic cancer cell survival and metabolism. Genetic depletion of autophagic components or pharmacological inhibition of autophagy with chloroquine can decrease tumorigenicity in vivo.^208,209^ Oncogenic *KRAS* can activate autophagy, and knockdown of *KRAS* expression or inhibition of its downstream MEK/ERK signaling cascade further induces autophagy and decreases both glycolytic and mitochondrial functions. Combined autophagy inhibition by hydroxychloroquine (HCQ) and MEK/ERK inhibitors showed enhanced antitumor activity in vivo.^209,210^ Although autophagy is usually regulated by nutrient and oxygen availability, pancreatic cancer cells often display high basal levels of autophagic flux even under nutrient-rich conditions.^211^ Pancreatic cancer cells use autophagy to obtain glucose and amino acids from catabolized substrates to fuel the citric acid cycle (TCA) cycle, OXPHOS, and ATP biosynthesis. It has been shown that autophagy induction in pancreatic cancer cells occurs as part of a broader transcriptional program regulated by the microphthalmia/transcription factor E (MiT/TFE) family of transcription factors.^212^ MiT/TFE-dependent autophagy–lysosome activation is required to maintain intracellular amino acid pools, and knockdown of the encoding genes strongly suppresses tumor progression.^212^ In vitro, autophagy inhibition leads to disrupted redox state, elevated DNA damage, and decreased levels of metabolic substrates, resulting in suppression of pancreatic cancer cell proliferation.^209,213^ Notably, the disrupted redox state and defective mitochondrial respiration caused by autophagy inhibition can be rescued by supplying antioxidants and TCA cycle intermediates.^213,214^ Autophagy is also a key regulator of immunogenicity in pancreatic cancer cells by virtue of its selective targeting of major histocompatibility complex class I (MHC-I) for degradation. Elevated autophagy is strongly correlated with increased immune evasion and reduced CD8^+^ T cell infiltration in pancreatic cancer.^215,216^

## The tumor microenvironment of pancreatic cancer

The tumor microenvironment in pancreatic cancer is highly immunosuppressive and is characterized by an abundant stromal response as a desmoplastic reaction. Accumulating research has highlighted the critical roles of the tumor microenvironment in maintaining pancreatic cancer development; hence, the tumor microenvironment acts as a key determinant by which this malignancy acquires therapeutic resistance to currently available treatment.

### The pancreatic stroma in pancreatic cancer

Pancreatic cancer is characterized by an extensive and dense fibrous stroma, and the stromal composition can account for up to 90% of the total tumor volume. The crosstalk between tumor cells and the stromal microenvironment is complex, and stromal elements regulate pancreatic cancer progression in a more complex manner. The paradoxical effects of the pancreatic cancer-associated stroma on pancreatic cancer cells, which include both tumor-promoting and tumor-suppressive regulations, might represent a target for new therapeutic strategies based on context-dependent stromal alterations.^217,218^

#### The composition of the pancreatic cancer stroma

Pancreatic cancer is known for its stroma/desmoplastic reaction, comprising a heterogeneous mass of cells, including PSCs, fibroblasts, immune cells, ECM, and soluble proteins such as cytokines and growth factors.^219^ The main types of stromal cells that contribute to tumor progression are PSCs, CAFs, TAMs, regulatory T cells (Tregs), and MDSCs.^219^ PSCs can be classified as quiescent PSCs (qPSCs) or activated PSCs (aPSCs) depending on their activation state. As lipid storage cells in the pancreas, qPSCs are located in surrounding perivascular regions or basolateral aspects of acinar cells.^220^ qPSCs can be activated under some environmental stress conditions, including inflammation, hypoperfusion, and oxidative stress, etc. aPSCs can transdifferentiate into myofibroblast-like phenotype, constituting a growth-permissive microenvironment in the tumor tissue.^221^ However, aPSCs are not permanently sustained because they can revert to their inactivate state under the influence of several factors, e.g., apoptosis, senescence, tissue regression, or recovery.^220^ Macrophages, a key class of innate immune cells, are derived from the mononuclear phagocyte system in the bone marrow, and they contribute to desmoplasia and immunosuppression of pancreatic cancer. TAMs can respond to environmental signals (e.g., inflammatory cytokines IL-8, IL-6, IL-1β, IL-10),^222^ and it is thought that a high number of infiltrating TAMs may correlate with tumor size, prognosis, and patient survival in pancreatic cancer.^223^ Forkhead box P3-positive (Foxp3^+^) Tregs are a subtype of T cells with significant roles in immunological self-tolerance maintenance and immunosuppression modulation during tumor progression. Foxp3^+^ Tregs have been shown to interact with tumor-associated CD11c^+^ dendritic cells and to restrain the immunogenic activation of CD8^+^ T cells.^224^ In pancreatic cancer, Foxp3^+^ Tregs are an essential source of TGF-β ligands, promoting the differentiation of SMA^+^ fibroblasts (myCAF) for tumor progression. Furthermore, Foxp3^+^ Treg depletion results in the differentiation of inflammatory fibroblast subsets, which in turn drive increased myeloid cells recruitment and impair the alleviation of immunosuppression.^225^

MDSCs are a heterogeneous population of immature myeloid cells that can be found in both tumors and the spleen. MDSCs usually participate in various pathological processes, including tissue inflammation and tumor progression. MDSCs can build crosstalk with TAMs, Tregs, and other immune cells to suppress effector T cells in the tumor microenvironment.^226^ Pancreatic tumors can create a hypoxic environment that recruits the MDSCs to the tumor microenvironment where they exert their immunosuppressive effect.^227^

#### The origin and functions of CAFs in pancreatic cancer

CAFs are one of the most prominent and active components of the desmoplastic stroma of pancreatic cancer. They possess diverse functions, including a robust fibro-inflammatory stromal effect and extensive reciprocal crosstalk with pancreatic cancer cells.^228^ The exact origin of CAFs is unclear at this time because they are derived from various types of cells, e.g., adipocytes, epithelial cells, resident fibroblasts, and bone marrow-derived mesenchymal stem cells.^221^ The general consensus is that aPSCs are one of the predominant origins of CAFs in the pancreatic cancer stroma.^229^ Moreover, CAFs activation involves multiple complex pathways and cytokines, including EMT, the sonic hedgehog (SHH) pathway, and various cytokines, including IL-1, IL-6, IL-10, TGF-β, and tumor necrosis factor (TNF-α).^228,230^

As major players in pancreatic tumorigenesis, activated CAFs from PSCs have numerous anti- and pro-tumorigenic influences on cancer cells in the tumor microenvironment. CAFs establish signal communication with tumor cells through a variety of mechanisms, e.g., EVs and classical paracrine.^221^ CAFs can increase the pancreatic cancer aggressiveness via EVs crosstalk that carries the annexin A6 (ANXA6)/LDL receptor-related protein 1/thrombospondin 1 complex under physiopathologic conditions (macrophages invasion, hypoxia, and lipid starvation).^231^ The presence of ANXA6^+^ EVs in the peripheral blood is restricted to pancreatic cancer patients, establishing these EVs as a potential biomarker of pancreatic cancer aggressiveness.^231^ Moreover, CAF-derived EVs can inhibit the metabolic reaction of mitochondrial OXPHOS and increase glycolysis and Gln-dependent reductive carboxylation in pancreatic cancer cells.^232^ Under chemotherapy, exposure of pancreatic cancer to gemcitabine might increase the release of CAF-derived EVs, which might further enhance chemoresistance and tumor cell survival.^233^ High expression of fibroblast activation protein (FAP) in CAFs has been established as a key enhancer of PDAC progression. Genetic deletion of FAP in the KPC mouse model results in delayed primary tumor formation in mice bearing pancreatic cancer tumors, leading to improved survival. Currently, researchers are attempting to establish FAP as a precise molecular target for guiding pancreatic cancer therapy.^234^ CAFs can enhance aerobic glycolysis and secrete high-energy metabolites, including lactate and pyruvate.^235^ Moreover, it has been demonstrated that enhanced autophagy in CAFs contributes to the release of amino acids, which tumor cells can use as anabolic substrates.^236^ CAFs also supply amino acids, TCA cycle intermediates, and lipids to tumor cells via exosomes, thus promoting pancreatic tumor growth, especially under nutrient-limited conditions.^232^ Lysophosphatidylcholine released from CAFs can be converted into lysophosphatidic acid (LPA) by autotaxin within the tumor microenvironment, subsequently exerting mitogenic signals via binding with the LPA-receptor on the surface of tumor cells, to support tumor growth.^237^

#### CAF heterogeneity in pancreatic cancer

Due to differences in their sources of origination and activation mechanisms, CAFs display heterogeneity that is characterized by several subgroups with different phenotypes and functions in pancreatic cancer tissue. For a long time, CAFs were only considered tumor promoters in the tumor microenvironment of the pancreatic cancer cells based on their involvement in tumor cell proliferation and survival via facilitating tumor invasion, metastasis, and immunosuppression.^238,239^ For example, as one of the traditional CAF activation pathways, the SHH pathway has been targeted by genetic deletion or chemical inhibition for tumor suppression. However, some recent studies have found that tumors can become more aggressive and undifferentiated upon Shh deletion in a pancreatic cancer mouse model.^218,240^ This evidence suggests that CAFs might comprise several subsets that have different or even opposing effects on tumor progression. Biffi et al. have identified two CAFs subtypes with either myofibroblastic characteristics or inflammatory phenotypes, which they have named “myofibroblastic CAFs” and “inflammatory CAFs,” respectively. Despite these observations, the exact origins of CAFs and their roles in pancreatic cancer progression have not yet been fully explored.^84^ It has been reported that genetically regulated phenotypes of pancreatic cancer affect pancreatic cancer progression by increasing matricellular fibrosis and tissue tension.^241^ Tape et al. found that oncogenic *KRAS*^*G12D*^ engages heterotypic fibroblasts, which subsequently instigate reciprocal signaling in tumor cells.^242^

Classical therapeutic approaches that focus on the depletion of the stromal desmoplasia usually lead to a disappointing result. In light of the complexity of the pancreatic cancer microenvironment,^243^ future therapeutic approaches that target both the tumor and the stroma may be able to achieve a better outcome.

### Immunomodulation in pancreatic cancer

**Fig. 3:**
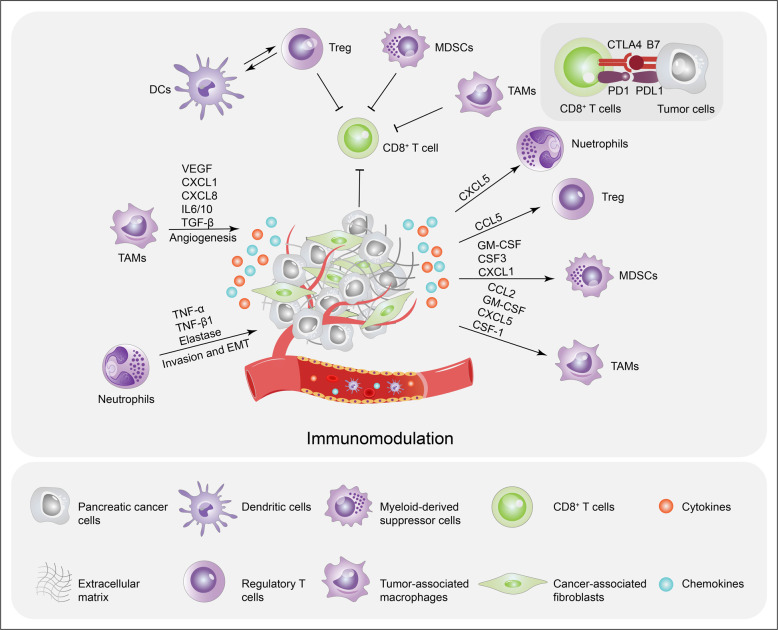
Immune evasion orchestrated by pancreatic cancer cells and stromal cells. The secretion and immunomodulation of pro-tumorigenic cytokines by pancreatic cancer cells and stromal cells are tightly regulated by oncogenic *KRAS*- or mutant *TP53*-dependent pathways. Pancreatic cancer cells secrete cytokines and, chemokines and recruit immunosuppressive cells, including MDSCs, TAMs, Treg cells, and neutrophils, which suppress the activity and functions of CD8^+^ cytotoxic T cells. Pancreatic cancer cells also evade the immune system by expressing PDL-1 to promote CD8^+^ T cell exhaustion. Immune cell infiltration also releases cytokines and growth factors that directly stimulate tumor growth by promoting angiogenesis and increasing the invasive ability of pancreatic cancer cells

Pancreatic cancer is often considered to be immunosuppressive based on a massive infiltration of immunosuppressive leukocytes and minimal antitumor T cell infiltration.^244^ Pancreatic cancer cells have been shown to cooperate with other regulatory immune cells to evade immune surveillance and to resist the cytotoxic effect of T lymphocytes (Fig. 3).^245^ Previous studies have shown that there is a correlation between the clinical significance and the composition and quantity of tumor-infiltrating immune cells and tumor-associated myofibroblasts.^246^ Mahajan et al. described a novel prognostic signature comprising distinct immune cell and stromal components for risk assessment of pancreatic cancer patients and prediction of their progression-free survival (PFS).^247^ Research has also shown that patients with higher levels of CD4^+^ and/or CD8^+^ T cells have significantly longer survival.^248^

The immunosuppressive tumor microenvironment of pancreatic cancer can be largely attributed to tumor cell-intrinsic pathways. Oncogenic *KRAS* mutations and KRAS-induced colony-stimulating factor (CSF) 2 (GM-CSF) production, promote pancreatic neoplasia progression via an influx of CD11b^+^Gr1^+^ immunosuppressive cells and suppressed antitumor T cell function.^249^ PI3K is a critical downstream effector of KRAS. One of its catalytic subunits, phosphatidylinositol-4,5-bisphosphate 3-kinase catalytic subunit alpha, has been shown to reduce the expression of MHC class I and CD80 in pancreatic cancer cells, leading to limited T cell recognition and pancreatic cancer cell clearance.^250^ Moreover, p53 and KRAS can cooperatively target the ADP ribosylation factor 6-ArfGAP with SH3 domain, and ankyrin repeat and PH domain 1 pathway to promote pancreatic cancer immune evasion, PD-L1 dynamics, and malignancy.^251^ Loss of p53 function activates JAK2-STAT3 signaling, which induces macrophage and neutrophil infiltration, reduces CD8^+^ T cell levels, and promotes tumor growth in pancreatic tumors in mice.^252^ In neoplastic pancreatic cancer cells, focal adhesion kinase (FAK) activity is hyperactivated and is correlated with high levels of TAMs, MDSCs, and Treg cells and low CD8^+^ cytotoxic T cell infiltration.^253,254^ In addition, FAK inhibition increases immune surveillance by mitigating the immunosuppressive pancreatic cancer tumor microenvironment, thereby rendering tumors responsive to immunotherapy.^254^ Similarly, inhibition of endogenous Myc triggers ubiquitous regression of pancreatic tumors by decreasing the levels of neutrophil and macrophage infiltration.^255^ Studies in a pancreatic cancer mouse model have demonstrated that Myc activation alone is sufficient to trigger the release of instructive signals that cooperatively coordinate changes in multiple immune and stromal cell types.^256^ Moreover, Myc and KRAS^G12D^ cooperatively regulate gene expression resulting in natural killer cell-mediated immune surveillance.^257^

Aside from genetic alterations that regulate the tumor microenvironment, pancreatic cancer can adopt other strategies of immune evasion, including the production of metabolites and immunosuppressing chemokines and cytokines. Lactate is robustly exported from pancreatic cancer cells via elevated monocarboxylate transporter 4 (MCT4)4 expression, and neighboring tumor cells in normoxic regions overexpress the lactate importer MCT1 to fuel OXPHOS and promote tumor growth.^168,258^ Furthermore, lactate secreted from pancreatic cancer cells remodels the tumor microenvironment and contributes to the M2-like polarization of TAMs, which leads to suppressed immunity.^259,260^ TAMs participate in metabolic crosstalk and elevate the glycolytic signature, which in turn promotes pancreatic cancer vascularization and metastasis.^261^ TAM-secreted C–C motif chemokine ligand 18 (CCL18) interacts with ITPNM family member 3 to induce vascular cell adhesion molecule-1 overexpression in pancreatic cancer cells, thereby promoting lactate production accompanied by elevated aerobic glycolysis. This effect activates macrophages into a TAM-like phenotype, forming a positive feedback loop.^259^ Pancreatic cancer cells can secrete CCL5 to promote the recruitment of Treg cells into the tumor site.^262,263^ The levels of tumor-derived GM-CSF,^249^ M-CSF,^249^ CSF1,^264^ CCL2, and CXCL5^265,266^ are often highly elevated by mutant *KRAS* expression and are associated with TAM infiltration. The chemokine CCL2, which can be produced by pancreatic cancer cells, facilitates the recruitment of immunosuppressive C–C motif chemokine receptor 2-positive TAMs into the tumor microenvironment, thereby limiting T cell infiltration.^267,268^ CXCL5 is an important attractant for immune cell accumulation. Pancreatic cancer patients with high CXCL5 expression have more intratumoral M2-polarized macrophages, neutrophils, and IgG^+^ plasma cells than those with low CXCL5 expression.^269^ Studies have demonstrated that tumor-derived CXCL1,^270^ GM-CSF,^271^ and CSF-3^272^ mediate the recruitment of MDSCs to the tumor microenvironment. CXCL1 has been shown to facilitate the recruitment of myeloid cells and inhibition of the T cell infiltration into tumors.^273^ Moreover, CSF1 serum levels are upregulated in pancreatic cancer, and higher levels correlate with increased macrophage infiltration and advanced tumor stage.^274,275^ Pancreatic cancer cells also secrete various immunosuppressive cytokines, including TGF-β and IL-10, which (1) coordinate immunosuppressive tumor microenvironment formation and (2) recruit cells involved in immune evasion, e.g., TAMs and Treg cells, to evade antitumor immunity.^276,277^ Foxp3 expressed by pancreatic cancer cells can activate CCL5 to promote the recruitment of Treg cells from peripheral blood to the tumor site, which negatively correlates with poor prognosis in pancreatic cancer.^262^ It has been demonstrated that neutrophils can increase pancreatic cancer metastasis by releasing a collection of cytokines, including TNF-α, TGF-β1, and elastase.^278,279^ Moreover, increased PD-L1 expression on pancreatic cancer cells can restrain T cell function by promoting CD8^+^ T cell exhaustion. Pancreatic cancer can even induce CD8^+^ T cell apoptosis via a Fas/FasL counterattack.^280,281^

## Clinical perspectives of current advances in pancreatic cancer

### Novel biomarkers of pancreatic cancer based on liquid biopsy

Currently, pancreatic cancer detection and diagnosis primarily rely on imaging modalities, including transabdominal ultrasonography, computed tomography (CT), magnetic resonance imaging), positron emission tomography, endoscopic retrograde cholangio-pancreatography, and endoscopic ultrasonography.^282^ These screening technologies have limitations in their detection capability. For instance, they are ineffective at detecting early-stage pancreatic cancer and small metastases or peritoneal lesions, etc.^283,284^ Serum tumor markers such as carcinoembryonic antigen and carbohydrate antigen have been widely used for pancreatic cancer diagnosis in clinical settings. However, they lack sensitivity and specificity as pancreatic cancer biomarkers.^285,286^ To date, novel markers such as ncRNAs (miRNA and lncRNA),^287,288^ genetic markers (e.g., *KRAS*, *TP53*, *SMAD4*, and *CDKN2A*),^17,289^ circulating tumor DNA (ctDNA),^290^ circulating tumor cells (CTCs),^291,292^ and exosomes^293,294^ have been explored. They show great potential for pancreatic cancer early detection, and might provide significant improvement in the future management and treatment outcome of pancreatic cancer. A liquid biopsy allows for serial, real-time monitoring of the dynamics and complex nature of cancer. In this section, we focus on the most promising liquid biopsy markers: CTCs, ctDNA, and exosomes, and their clinical applications in pancreatic cancer.

CTCs, which can be found in pancreatic cancer patients’ circulation, provide useful information for diagnosis, staging, and prognosis, and they even can act as novel personalized treatment targets.^295,296^ Some studies have reported that CTCs can be detected and used as an early-stage diagnostic marker using a number of methods, including negative enrichment, immunofluorescence and in situ hybridization (FISH) of chromosome (NE-iFISH) system, and subtraction enrichment and immunostaining-fluorescence in situ hybridization (SE-iFISH). NE-iFISH exhibits a very high CTC detection rate (90%) in pancreatic cancer patients. SE-iFISH has both high sensitivity (88%) and high specificity (90%) in pancreatic cancer with a cutoff value of 2 cells/7.5 ml.^297,298^ CTCs in venous blood samples from pancreatic cancer patients were evaluated using a microfluidic NanoVelcro CTC chip and CTCs were detected in 54 out of 72 patients with a sensitivity and specificity of 75.0% and 96.4%, respectively.^299^ Despite differences in CTCs enrichment and detection methods, studies have consistently shown that a higher number of CTCs could be predictive of reduced survival.^297,300–302^ In addition, a higher number of CTCs in the portal vein correlated well with a higher risk of cancer recurrence and liver metastases after surgery. For example, at a 3-year follow-up with patients who underwent surgical resection, portal vein CTC-positive patients presented a higher rate of liver metastases than CTC-negative patients.^303^ In another study, 84.6% of patients with a high portal CTCs count (defined as >112 CTCs/2 ml blood) developed liver metastases within 6 months after surgery.^304^ CTCs can also be a promising biomarker or useful for monitoring the response to chemotherapy of pancreatic cancer patients. It has been reported that triploid CTCs (which show three hybridization signals for a given chromosome) <3 groups displayed significantly increased 1-year survival and OS compared with the triploid CTCs ≥3 group.^298^ Moreover, advanced pancreatic cancer patients can be tested for the presence of circulating tumor microemboli, whose presence predicts a low response to chemotherapy and poor survival.^298,305^

ctDNA, which represents a small fraction of the total cell-free DNAs (cfDNA), is derived from either the CTCs or the tumor cells.^306^ The sensitivity of ctDNA detection is generally lower than that of CA19-9 detection for early pancreatic cancer diagnosis.^307^ However, ctDNA proved to be a more suitable biomarker than CA19-9 and CTCs for the prognosis for pancreatic cancer patients. The higher burden of *KRAS* mutation on cfDNA levels has been shown to be negatively correlated with OS.^308–310^ Furthermore, the ctDNA level is a good indicator of pancreatic cancer progression during or after treatment (chemotherapy and/or surgery). Pancreatic cancer patients with detectable ctDNA showed poorer OS and a higher risk of cancer recurrence compared with those with undetectable ctDNA.^37,311–313^

Tumor-derived exosomes are enriched with DNA, mRNA, miRNA, and protein both inside and on their surface, establishing them as potential biomarkers for early-stage diagnosis of pancreatic cancer.^314–316^ Melo et al. reported that the biomarker glypican-1 (GPC1) was specifically enriched on pancreatic cancer exosomes.^317^ GPC1(+) GPC1-circulating exosomes were detected in the serum of pancreatic cancer patients with 100% specificity and sensitivity, thereby distinguishing pancreatic cancer patients from patients with nonmalignant pancreatic disease and healthy subjects.^317–319^ In addition, certain exosomal miRNAs (miR-10b, -21, -30c, -181a, and -let7a) had 100% sensitivity and 100% specificity, and ephrin receptor A2-EV had 94% sensitivity and 85% specificity in distinguishing pancreatic cancer from healthy controls.^320,321^ With such high specificity and sensitivity, pancreatic tumor-derived exosomes might be one of the most promising markers for both early-stage pancreatic cancer diagnosis and monitoring of the therapeutic response during treatment.^321–324^

While liquid biopsies are increasingly being adopted for potential biomarkers for cancer diagnosis, prediction, and surveillance, there is a need for developments in assay technology for the isolation, quantification, and analysis of biomarkers based on liquid biopsy. For example, it is difficult to isolate tumor-specific DNA because of the limitations of the currently available techniques.^325^ Despite these shortcomings, significant research is still dedicated to liquid biopsies because of their potential advantages. One well-validated, Food and Drug Administration (FDA)-approved liquid biopsy test panel, MSK-IMPACT, was developed at the Memorial Sloan Kettering Cancer Center. They have also developed another liquid biopsy assay called the Analysis of Circulating Cell-free DNA to Evaluate Somatic Status.^326^ By using the latter panel, Razavi et al. identified cancer-derived somatic variants in plasma-circulating cfDNA and highlighted the importance of matched cfDNA white blood cell sequencing for accurate variant interpretation.^327^ This panel was also used to identify clinically relevant mutations and mutation signatures as well as novel noncoding alterations.^328^ This panel was subsequently expanded via Clinical Laboratory Improvement Amendment-certified testing for the analysis of tumor-derived and matched germline DNA samples, which has also been approved by the FDA.^329^ However, the majority of liquid biopsy assays still lack sufficient evidence of clinical validity and utility. Only after the demonstration of clinical validity and clinical utility, liquid biopsies may further reach their full potential and impact on the clinical management of pancreatic cancer patients.

### Subtype-specific therapeutic responses in pancreatic cancer

Pancreatic cancer is extensive heterogeneity amongst patients with respect to treatment response. Basal-like tumors have a higher frequency of *TP53* mutations,^36,46,48^ while the classical subtype is characterized by GATA binding protein 6 (*GATA6*) expression and *KRAS* dependency.^44,46^ Importantly, patients with basal-like tumors have higher pathologic grades and worse OS compared with those with classical tumors.^44,46,48,50,330^ Nevertheless, there is no consensus on subtype-specific treatment regimens. Although Collisson et al. reported that QM-PDA subtype patients had significantly worse outcomes compared with patients with other subtypes, they found that in pancreatic cancer cells, the QM-PDA subtype was more sensitive to gemcitabine. By contrast, classical subtype cell lines were more sensitive to erlotinib.^44^ One possible explanation for these findings is that in vitro 2D-cultured cell lines might not manifest expected bulk RNA-sequencing (RNA-seq(-based molecular signatures, as Moffitt et al. reported that traditional 2D-cultured cell lines lack the classical subtype.^47^ Moffitt et al. also demonstrated that patients with basal-like tumors showed a tendency to benefit more from adjuvant therapy, although the effect was not significant.^47^ In vivo patient-derived xenograft (PDX) models or 3D organoid-based approaches might be more effective tools for investigating subtype-specific treatment responses.^45^ Interestingly, by using an organoid-based model, Tiriac et al. found that the basal-like subtype was enriched in an oxaliplatin-nonsensitive group, while it was present at similar frequencies in gemcitabine-sensitive and nonsensitive groups.^45^

Recently, Aung et al. reported the results of the COMPASS trial, showing that basal-like subtype tumors showed a poorer response to first-line chemotherapy (1/12 vs. 13/38 had a partial response in the basal-like vs. classical subtype, respectively). Patients with classical subtype tumors treated with mFOLFIRINOX had the best PFS.^331^ More recently, Chan et al. devised a more comprehensive method for molecular subtyping and for developing subtype-specific treatment regimens in pancreatic cancer.^46^ They reclassified pancreatic cancer samples into five subtypes: basal-like A, basal-like B, hybrid, classical A, and classical B. Distinguishing the basal-like A, basal-like B, and hybrid subtypes from the former basal-like/squamous subtype allowed the detection of more subtle subtype-specific chemotherapy responses; furthermore, Chan et al. reported that the basal-like A subtype showed the poorest responses to gemcitabine-based and mFOLFIRINOX chemotherapies.^46^ Remarkably, single-cell RNA-seq data provided evidence of the coexistence of basal-like and classical expression signatures within a single cancer sample; furthermore, subtype switching, a rare event due to the outgrowth of minor clones driven by therapeutic selection and genetic instability, was observed.^46^ Additional evidence of intratumoral transcriptional heterogeneity in pancreatic cancer was found by Hayashi et al. via multiregional sampling.^332^ In addition, Er et al. demonstrated a squamous subtype-specific synergistic effect of Src and MEK1/2 inhibitors with gemcitabine in pancreatic cancer cell lines.^333^

Limited access to freshly frozen samples and cost-prohibitive issues have limited the use of transcriptomic-based subtyping. Puleo et al. evaluated the transcriptome of formalin-fixed paraffin-embedded pancreatic cancer samples and successfully identified basal-like and classical subtypes.^48^ Law et al. reported proteomic-based subtyping of pancreatic cancer liver metastases and identified four distinct pancreatic cancer subtypes, i.e., metabolic, progenitor-like, proliferative, and inflammatory.^330^ Pancreatic cancer patients with metabolic and progenitor-like subtypes showed significant benefit from FOLFIRINOX treatment.

Hayashi et al. and Kalimuthu et al. provided paradigms for integrating clinical morphological and histological features with transcriptomic expression profiles such that pancreatic cancer molecular subtypes can be predicted based on morphological classification.^332,334^ Hayashi et al. found that histological squamous features and glandular patterns were consistent with the RNA-seq-defined basal-like and classical subtypes, respectively.^332^ They also identified intratumoral heterogeneity in transcriptional subtypes and suggested a subclonal feature of pancreatic cancer subtypes, which was inconsistent with Chan et al.^46^ Kalimuthu et al. identified four morphological patterns that segregated into two components, i.e., gland-forming and non-gland-forming, that corresponded to the classical and basal-like subtypes.^334^ They even found that morphological pattern-based subgroups provide better prediction of clinical outcomes than transcriptional subtypes. Efforts have also been made to simplify the classification of pancreatic cancer samples using surrogate markers, e.g., *TP63*,^335^ *YAP1*,^92^ *HNF1 homeobox A*, *keratin 81* (*KRT81*),^50^ *GATA6*,^44,46^ and *KRT17.*^336^ Interestingly, Kaissis et al. reported that a machine learning-based analysis of preoperative CT images could predict molecular pancreatic cancer subtypes.^337^ These findings might facilitate clinical patient stratification and provide guidance for precision medicine.

Although some studies have demonstrated the feasibility of prospective genomic profiling of advanced pancreatic cancer, this approach is time-consuming and costly.^331,338^ In addition, clustering algorithms are threshold sensitive, and molecular classifiers are heavily confounded by limitations such as sampling methods, tumor cellularity, RNA quality, and tumor heterogeneity.^338,339^ Nevertheless, molecular subtyping now guides the optimization of therapeutic strategies and provides valuable opportunities for improving pancreatic cancer patient outcomes.

### Current preclinical and clinical developments in pancreatic cancer

#### Current preclinical therapeutic models in pancreatic cancer

Identification of clinically applicable approaches for pancreatic cancer treatment relies heavily on the availability of preclinical models. In recent decades, novel preclinical models, e.g., PDX systems and patient-derived organoids (PDO), have been developed and are becoming increasingly used in drug screening, biomarker development, and evaluation of personalized therapeutic strategies. PDX and PDO models have positive predictive values of 80 and 88% for patient-specific sensitivities to therapeutic agents, respectively.^340–342^ However, PDX and PDO models are primarily suitable for basic research and have severe limitations for clinical use. For example, it normally takes several months for a PDX model to yield preclinical results. Moreover, it is infeasible to study early carcinogenesis, progression, and tumor immunology based on PDX or PDO models.

Recently, advances in genetic engineering techniques and an increased understanding of the mechanisms of pancreatic oncogenesis have supported the development of GEMMs useful as pancreatic cancer preclinical models (Table 1). Most of the GEMMs currently used in translational oncological studies are Cre/loxP-based models. Endogenous expression of oncogenic *KRAS*^*G12D*^ induces PanIN in mice, and a subset of these mice develop pancreatic cancer tumors at an advanced age, revealing that additional events are necessary for tumor formation onset.^343^

**Table 1:** An overview of common genetically engineered mouse models of pancreatic cancer and their key characteristics

Genotype	Preneoplastic lesion	Cancer phenotype	Metastasis	Features	References
*Pdx1*; *KRAS*^*G12D/+*^	PanIN	PDAC	Yes	Long latency	^343^
*Ptf1a*^*+/Cre*^; *KRAS*^*G12D/+*^	PanIN	PDAC	Yes	Long latency	^343^
*KRAS*^*G12D/+*^; *Ela-TGFa*	PanIN	IPMN	Yes (50%)	Moderate latency	^350^
*Pdx1-Cre*; *LSL-KRAS*^*G12D/+*^; *Ink4a/Arf*^*lox/lox*^	PanIN	PDAC	Yes (21%)	Short latency and high penetrance	^346^
*Pdx1-Cre*; *LSL-KRAS*^*G12D/+*^; *LSL-TP53*^*R172H/+*^	PanIN	PDAC	Yes (63%)	Accelerated development of metastatic PDAC	^345^
*LSL-KRAS*^*G12D*^; *TP53*^*fl/fl*^ (in situ electroporation of Cre recombinase and myrAkt2)	PanIN	PDAC	Yes (>60%)	High efficiency, short latency	^384^
Co-electroporation of *SB13* transposase with a *KRAS*^*G12V*^-expressing transposon, Cre recombinase and myrAkt2 in *TP53*^*fl/fl*^ mice	PanIN	PDAC	Yes (>70%)	High efficiency, short latency	^384^
*Pdx1-Cre*; *KRAS*^*G12D/+*^; *Ink4a*^−^^*/−*^ *TP53*^*lox/lox*^	PanIN	PDAC	Yes (20%)	Short latency and high penetrance	^347^
*Pdx1-Cre*; *KRAS*^*G12D/+*^; *Smad*^*lox/lox*^	IPMN	PDAC	Yes (38%)	Model of IPMN to PDAC progression	^351,352^
*Ptf1a*^*+/Cre*^; *KRAS*^*G12D/+*^; *Smad*^*lox/lox*^	MCN	PDAC	Yes (18%)	MCNs resembling human disease	^385^
*Ptf1a*^*+/Cre*^; *KRAS*^*G12D/+*^; *ATM*^*lox/lox*^	PanIN	PDAC	Yes (78%)	High metastasis tendency	^348^

*IPMN* intraductal papillary mucinous neoplasm, *MCN* mucinous cystic neoplasm, *PanIN* pancreatic intraepithelial neoplasia, *PDAC* pancreatic ductal adenocarcinomas

Strains for targeting pancreatic progenitor cells, such as *Pdx1-Cre* transgenic or *Ptf1a*^+/Cre^ knock-in strains in the context of endogenous mutant *KRAS*^*G12D*^ (generally referred to as KC mice) have been successfully used to recapitulate the development and progression of PanIN lesions to pancreatic cancer.^343,344^ Furthermore, the KPC PDAC mouse model, first described in 2005, allows conditional activation of the *KRAS*^*G12D*^ mutation and a *TP53* mutation (*Pdx1-Cre*; *LSL-KRAS*^*G12D/+*^; *LSL-TP53*^*R172H/+*^) via Cre-Lox technology.^345^ In addition, combined with the *KRAS*^*G12D*^ mutation, *Ink4a/Arf* deficiency (*Pdx1-Cre*; *LSL-KRAS*^*G12D/+*^; *Ink4a/Arf*^*lox/lox*^), *ATM* deficiency (*Ptf1a*^*+/Cre*^; *KRAS*^*G12D/+*^; *ATM*^*lox/lox*^), or a *TP53*^*lox/lox*^ background plus *P16*^*Ink4a*^ deficiency (*Pdx1-Cre*; *KRAS*^*G12D/+*^; *Ink4a*^*−/−*^ *TP53*^*lox/lox*^) result in accelerated progression from PanIN to invasive PDAC.^345–349^ Concomitant expression of TGFα and *KRAS*^*G12D*^ leads to the development of cystic papillary lesions that resemble human IPMN (*KRAS*^*G12D/+*^; *Ela-TGFa*).^350^ Concomitant presence of *KRAS*^*G12D*^ and *SMAD* deficiency (*Pdx1-Cre*; *KRAS*^*G12D/+*^; *Smad*^*lox/lox*^ or *Ptf1a*^*+/Cre*^; *KRAS*^*G12D/+*^; *Smad*^*lox/lox*^) leads to the development of IPMN or mucinous cystic neoplasm, respectively.^351,352^ Compared with traditional xenograft models, these GEMMs more faithfully recapitulate key morphological and molecular PDAC features. They can be used to study early carcinogenesis, progression, and tumor immunology of traditional xenograft models. GEMM models also offer higher predictive value for clinical diagnostic and therapeutic interventions. However, despite the increasing repertoire of animal models that emulate key genetic driver mutations found in pancreatic cancer, it is important to emphasize that many GEMMs do not fully recapitulate all aspects of the clinical population by fully covering the full range of genetic diversity and metastatic spread, as demonstrated by multiple failed clinical trials.^353,354^

#### Clinical strategies in pancreatic cancer

For pancreatic cancer patients, the only potentially curative choice is surgical resection of the pancreas. This approach is restricted to 20% of cases with anatomically resectable disease. Moreover, up to 50% of patients have incomplete resection accompanied by positive surgical margins. For those patients, the overall 5-year survival sharply decreases to 7%.^16,355,356^ It has been demonstrated that adjuvant chemotherapy can improve OS, but postoperative complications limit the intended treatment in 50% of patients.^357^ For patients with borderline or locally advanced pancreatic cancer, neoadjuvant therapy can convert unresectable disease into a potentially resectable state, which benefits OS.^358^

**Fig. 4:**
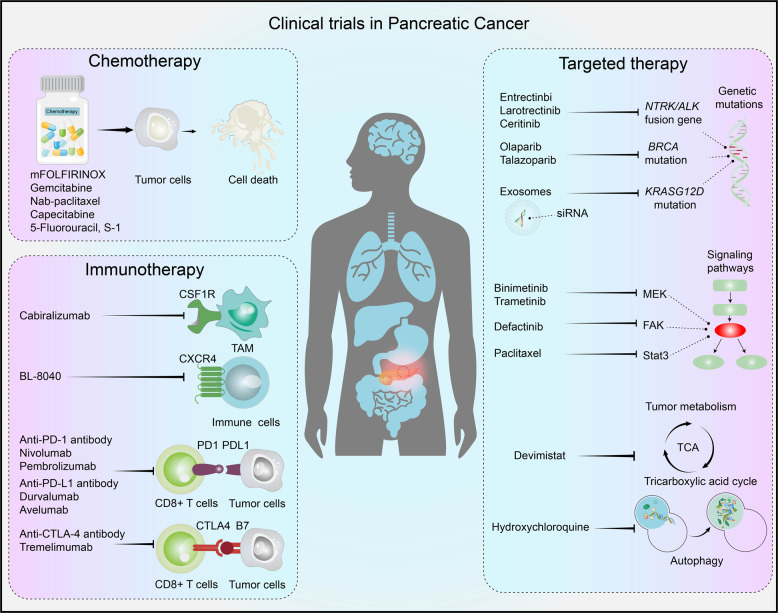
Current clinical strategies in pancreatic cancer. For patients with pancreatic cancer, the primary clinical strategies rely on chemotherapy, whereas novel therapeutic agents targeting DNA repair, gene mutations, tumor metabolism, tumor microenvironments, or immune checkpoints might improve their prognosis. Currently, increasing interest has emerged in combined chemotherapy and immunotherapy or targeted therapy

Current guidelines recommend FOLFIRINOX, mFOLFIRINOX, gemcitabine, or gemcitabine plus nab-paclitaxel for preoperative pancreatic cancer treatment. From 1997 to 2011, the first-line chemotherapy for patients with advanced pancreatic cancer was gemcitabine monotherapy.^10^ The treatment method changed in 2011 when FOLFIRINOX demonstrated a better survival benefit compared with that of gemcitabine monotherapy.^11^ Thereafter, gemcitabine plus nab-paclitaxel was adopted as another first-line treatment option. This treatment method had a better survival benefit (median OS 8.5 vs. 6.7 months, hazard ratio 0.72, 95% confidence interval 0.62–0.83, *P* < 0.001) compared with gemcitabine monotherapy.^12^ According to a large-scale retrospective study, FOLFIRINOX and nab-paclitaxel plus gemcitabine have similar outcomes.^359^ However, these therapeutic methods are reserved for otherwise healthy patients with good performance status. For the elderly and patients with a poor performance status, gemcitabine monotherapy is still considered a more tolerable treatment.^360^ The updated pancreatic cancer treatment guidelines recommend pembrolizumab immunotherapy for patients that carry mismatch repair (MMR) deficiencies or microsatellite instability after the failure of first-line therapies.^361^ As a second-line treatment option for pancreatic cancer, a combination of 5-FU and liposomal irinotecan is the only approved therapeutic method.^362^ In addition to these cytotoxic chemotherapies, novel agents are also under active investigation. The addition of leucovorin to S-1 improved PFS in patients with gemcitabine-refractory advanced pancreatic cancer.^363^ For patients with pancreatic cancer, novel therapeutic trials targeting DNA repair, gene mutations, tumor metabolism, the tumor microenvironment, or immune checkpoints might improve their prognosis (Fig. 4 and Table 2).

**Table 2:** Ongoing clinical trials for pancreatic cancer treatment

Condition or disease	Intervention/treatment	Target	ClinicalTrials.gov identifier	Phase	Status and results	Reference
Metastatic PDAC	Mesenchymal stromal cell-derived exosomes with *KRAS*^*G12D*^ siRNA	KRAS	NCT03608631	1	Not yet recruiting	^365^
NTRK fusion-positive solid tumors	Entrectinbi	NTRK inhibitor	NCT02568267	2	Recruiting	^366^
NTRK fusion-positive solid tumors	Larotrectinib	NTRK inhibitor	NCT02576431	2	Recruiting	^367^
Advanced solid tumors	Ceritinib	ALK inhibitor	NCT02227940	1	Completed	
*BRCA*-mutated metastatic pancreatic cancer	Olaparib maintenance	PARP inhibitor	NCT02184195	3	Completed, ORR and PFS improved	^14^
Advanced PDAC	Nivolumab with cabiralizumab with or without chemotherapy	Anti-PD-1 antibodies and CSF-1R antibodies	NCT03336216	2	Active, not recruiting	
Metastatic pancreatic cancer	Pembrolizumab and BL-8040	Anti-PD-1 antibodies and CXCR4 antagonist	NCT02907099	2	Active, not recruiting	
Non-colorectal *MSI-H/dMMR*-deficient cancer	Pembrolizumab	anti-PD-1 antibodies	NCT02628067	2	Completed, ORR 18.2%, CR 4.5%, and PR 13.6% for PDAC	^373^
Metastatic PDAC	Durvalumab with or without tremelimumab	Anti-PD-L1 antibodies, anti-CTLA-4 antibodies	NCT02558894	2	Completed, tolerated, ORR 3.1% for combination therapy	
Locally advanced or metastatic *RAS*-mutant solid tumors	Avelumab, binimetinib, and talazoparib, second line	Anti-PD-L1 antibodies, MEK inhibitor, PARP inhibitor	NCT03637491	2	Recruiting	
Metastatic PDAC	Devimistat with mFOLFIRINOX	Tricarboxylic acid cycle modulation	NCT03504423	3	Recruiting	^378^
Advanced or metastatic PDAC	Hydroxychloroquine with gemcitabine/nab-paclitaxel	Autophagy inhibitor	NCT01506973	1/2	Active, not recruiting	
Advanced or metastatic PDAC	Hydroxychloroquine and trametinib	Autophagy inhibitor, MEK inhibitor	NCT03825289	1	Recruiting	
Advanced or metastatic PDAC	Paclitaxel liposome and S-1	–	NCT04217096		Active, not recruiting	
Stage IV untreated PDAC	PEGPH20 based on nab-paclitaxel and gemcitabine	Recombinant human hyaluronidase	NCT01839487	2	Completed, improved PFS and ORR	^375^
Previously treated Hyaluronan high metastatic PDAC	PEGPH20 with pembrolizumab	Recombinant human hyaluronidase, anti-PD-1 antibodies	NCT03634332	2	Recruiting	
Advanced solid tumors	Defactinib with pembrolizumab	FAK inhibitor, PD-1 antagonist	NCT02758587	1/2	Recruiting	
PDAC	GSK2256098 with trametinib	FAK inhibitor, MEK inhibitor	NCT02428270	1	Active, not recruiting	
Unresectable pancreatic cancer	Pamrevlumab based on gemcitabine and nab-paclitaxel	CTGF antagonism	NCT02210559	1/2	Active, not recruiting	
Pancreatic cancer	CAR T cells, second line		NCT03323944	1	Active, not recruiting	
Metastatic pancreatic cancer	Paclitaxel and gemcitabine plus or without napabucasin	STAT3 and cancer cell stemness inhibitor	NCT03721744	3	Recruiting	
Metastatic PDAC	Napabucasin plus nab-paclitaxel with gemcitabine	Cancer stemness inhibitor	NCT02993731	3	Active, not recruiting	

*CR* complete response, *ORR* objective response rate, *PARP* poly-(ADP-ribose) polymerase, *PDAC* pancreatic ductal adenocarcinomas, *PFS* progression-free survival, *PR* partial response

The high prevalence of *KRAS* mutations (carried by >90% of pancreatic cancer patients) has led to considerable interest in KRAS-targeted therapies. Unfortunately, current approaches for direct targeting of mutant KRAS protein are ineffective due to their high affinity for GTP and/or GDP.^36,364^ An alternative strategy was developed to target KRAS by using exosomes or small EVs loaded with small interfering RNAs that target *KRAS*^*G12D*^. This study has entered a phase 1 clinical trial for patients with metastatic pancreatic cancer (NCT03608631).^365^ Furthermore, the National Cancer Institute in the United States also established the RAS Initiative in 2013 to explore effective therapies for RAS-related cancers (https://www.cancer.gov/research/key-initiatives/ras).

Increasing interest has also emerged in the targeting of low-prevalence, actionable aberrations, including BRCA1/2, NTRK1/2/3, or MMR deficiencies. In three phase 1 and 2 clinical trials evaluating the treatment of patients with advanced or metastatic NTRK fusion-positive solid tumors with a potent TRK inhibitor, entrectinib, a complete response (CR) of 7% and a partial response (PR) of 50% were achieved (NCT02097810, NCT02568267, EudraCT 2012-000148-88).^366^ In other clinical trials evaluating the efficacy of the TRK inhibitor larotrecitinib, the objective response rate (ORR) reached 75% among 55 patients with NTRK fusion-positive tumors, including one pancreatic cancer patient who had a PR (NCT02122913, NCT02637687, NCT02576431).^367^ Larotrectinib and entrectinib received FDA approval for use as tissue-agnostic indicators in patients with solid tumors with NTRK fusions in November 2018 and August 2019, respectively. Furthermore, the use of ALK inhibitors in pancreatic cancer patients is currently under investigation. ALK rearrangements have also been established as a promising molecular target in other malignancies, e.g., non-small cell lung cancer.^368^ In a 2017 study, five cases harboring an ALK fusion gene were identified via comprehensive genomic profiling of 3170 pancreatic cancer patients. Among them, four patients were treated with an ALK inhibitor, three of which showed a stable radiographic response and/or normalization of serum CA19-9.^369^ A phase 1 study to combine ceritinib, a novel ALK inhibitor, with chemotherapy for the treatment of advanced pancreatic cancer has just completed (NCT02227940).

Therapeutic strategies based on BRCA-poly (ADP-ribose) polymerase (PARP) synthetic lethality have shown to be effective for the treatment of patients with BRCA1/2 mutations. A multicenter phase 2 trial of olaparib (a PARP inhibitor) for the treatment of patients with a germline BRCA1/2 mutation and recurrent cancer achieved an ORR of 26.2% overall and an ORR of 21.7% for pancreatic cancer (NCT01078662).^370^ In the POLO trial and a randomized phase 3 trial for patients with metastatic pancreatic cancer with a germline BRCA1/2 mutation after at least 16 weeks of platinum-based chemotherapy, olaparib as a maintenance therapy achieved an ORR of 20% with a PFS median of 7.4 months, compared with 10% and 3.8 months in the placebo group (NCT02184195).^14^

The tumor microenvironment is another area of interest in pancreatic cancer therapeutic exploration. Immune checkpoint inhibitors (ICIs) have emerged as a new treatment paradigm for patients with certain types of tumors. However, the results of early clinical trials investigating the efficacy of the anti-CTLA-4 antibody ipilimumab or the anti-PD-L1 antibody BMS-936559 in patients with advanced pancreatic cancer were disappointing.^371,372^ In the phase 2 KEYNOTE-158 study of pembrolizumab (an anti-PD-1 monoclonal antibody), an ORR of 34.3% was achieved in patients with previously treated, advanced non-colorectal high microsatellite instability (MSI-H)/DNA MMR (dMMR)-deficient cancer, and in pancreatic cancer patients, an ORR of 18.2%, a CR of 4.5%, and a PR of 13.6% were achieved (NCT02628067).^373^ Based on these results, the FDA granted accelerated approval to pembrolizumab for adult and pediatric patients with unresectable MSI-H or dMMR solid tumors in May 2017. Clinical trials of PD-1 blockade or PD-L1 combined with other ICIs or blockade are currently ongoing (NCT03336216, NCT02907099, NCT02558894, NCT03637491). Hyaluronic acid is a hydrophilic glycosaminoglycan whose overproduction in the stroma of patients with pancreatic cancer leads to increased interstitial tumor pressure, reducing tumor perfusion and access of anticancer drugs to the tumor.^374^ A phase 2 trial showed that the addition of PEGPH20 (pegvorhyaluronidase-α, recombinant human hyaluronidase) to nab-paclitaxel and gemcitabine resulted in significant improvements in ORR (45 vs. 31%) and PFS (9.2 vs. 5.2 months) in pancreatic cancer patients with high hyaluronic acid expression levels (NCT01839487).^375^ However, a later phase 3 study failed to show improvements in OS or PFS, and the results did not support additional development of PEGPH20 as a treatment for metastatic PDAC, as a high rate of dose holds and reductions in the PEGPH20 arm might have led to lower chemotherapy drug exposure, which could have contributed to the inferior survival outcomes with PEGPH20.^376^

Metabolic reprogramming can also be a therapeutic target in pancreatic cancer. Devimistat, which inhibits enzymes in the mitochondrial tricarboxylic acid cycle, is hypothesized to act synergistically with cytotoxic agents to induce decreased production of anabolic intermediates required for DNA damage repair. A phase 1 study of devimistat combined with mFOFIRINOX for the treatment of metastatic pancreatic cancer patients showed an ORR of 61%, including a CRR of 17% (NCT01835041).^377^ Additional clinical trials evaluating the safety and efficacy of devimistat combined with gemcitabine and nab-paclitaxel or mFOLFIRINOX are ongoing (NCT03435289, NCT03504423).^378^ Inhibition of autophagy in pancreatic cancer primary tumors contributes to a metabolic defect that leads to decreased mitochondrial OXPHOS and significant growth suppression.^213^ In a phase 2 study, autophagy inhibition using HCQ in patients with metastatic pancreatic cancer failed to achieve therapeutic efficacy.^379^ Further clinical trials to test the effectiveness of HCQ combined with cytotoxic agents or MEK inhibitors are undergoing (NCT01506973, NCT03825289).

A number of combined therapeutic approaches have already been introduced. Preclinical research has shown that FAK inhibition can lead to decreased levels of fibrosis, synergy with chemotherapy and ICIs, and improved survival outcomes in the KPC mouse model of pancreatic cancer.^254^ Currently, a number of clinical trials to investigate the efficacy of combinations of FAK inhibitors and ICIs and/or cytotoxic agents or MEK inhibitors are ongoing (NCT02546531, NCT02758587, NCT02428270). Connective tissue growth factor (CTGF), a member of the CCN family of secreted proteins, is involved in ECM production, desmoplasia, and tumor progression.^380^ Preclinical data indicated that CTGF antagonism with the therapeutic monoclonal human antibody pamrevlumab enhanced the response to gemcitabine in pancreatic cancer mouse models.^381^ The safety and efficacy of the agent in combination with gemcitabine and nab-paclitaxel for the treatment of patients with unresectable pancreatic cancer were evaluated in a phase 1/2 trial (NCT02210559). The results indicated that the addition of pamrevlumab to neoadjuvant therapy in locally advanced pancreatic cancer patients might lead to higher resectability and resection rate. Currently, two phase 3 studies to investigate the efficacy of the addition of napabucasin to gemcitabine and nab-paclitaxel for treating metastatic pancreatic cancer patients are ongoing (NCT03721744, NCT02993731).^382^

Preclinical investigations in pancreatic cancer PDX models identified a small subset of tumor cells (termed CSCs) that determines self-renewal and metastatic phenotypes of pancreatic cancer.^383^ A phase 1b/2 study of the cancer stemness inhibitor napabucasin combined with gemcitabine and nab-paclitaxel for treating metastatic pancreatic cancer achieved a CR of 3% and a PR of 42%.

In summary, for patients with resectable pancreatic cancer, mFOLFIRINOX and nab-paclitaxel plus gemcitabine regimens were shown to achieve the longest median OS. For patients with advanced or metastatic pancreatic cancer, treatment regimens based on the identification of potentially actionable alterations in small subsets of patients via comprehensive genomic profiling and monitoring of the efficacy of therapeutic strategies are currently being explored. The POLO trial, which involved the identification of germline BRCA mutations and targeted therapy, proved to be effective, and such approaches are worth further exploration.

## Conclusions and future perspectives

Pancreatic cancer remains one of the most common and deadly cancers with limited options for effective therapy. Meaningful clinical progress in diagnostic investigations, surgical techniques, and systemic therapies are certain to improve pancreatic cancer patient survival. A deeper understanding of the biology and genetics of pancreatic cancer, including new insight into driver gene mutations, tumor metabolism, and the tumor microenvironment, might lead to promising and innovative therapeutic strategies. It has been widely proposed that targeting a single molecule or pathway is unlikely to yield more pancreatic cancer therapies. Both subtype-specific therapy and combined therapy might represent more promising strategies to control tumor progression. Recent advances showing that pancreatic cancer patients with germline BRCA mutations benefit from PARP inhibitors might inspire novel strategies that further increase the clinical efficacy of subtype-specific therapy. While clinical progress has never been clearer in the improvement of outcomes in pancreatic cancer patients, the path to clinical translation of novel therapeutic approaches will be greatly enhanced by the use of more sophisticated animal models and multidisciplinary clinical collaborations.
